# Supplementary figures and images for: Gelatinase regulates the egress of intracellular replicating populations during Enterococcus faecalis infection
Source: PLoS Pathog. 2026 Mar 10;22(3):e1013738. doi: 10.1371/journal.ppat.1013738 (PMC12994788; doi:10.1371/journal.ppat.1013738)

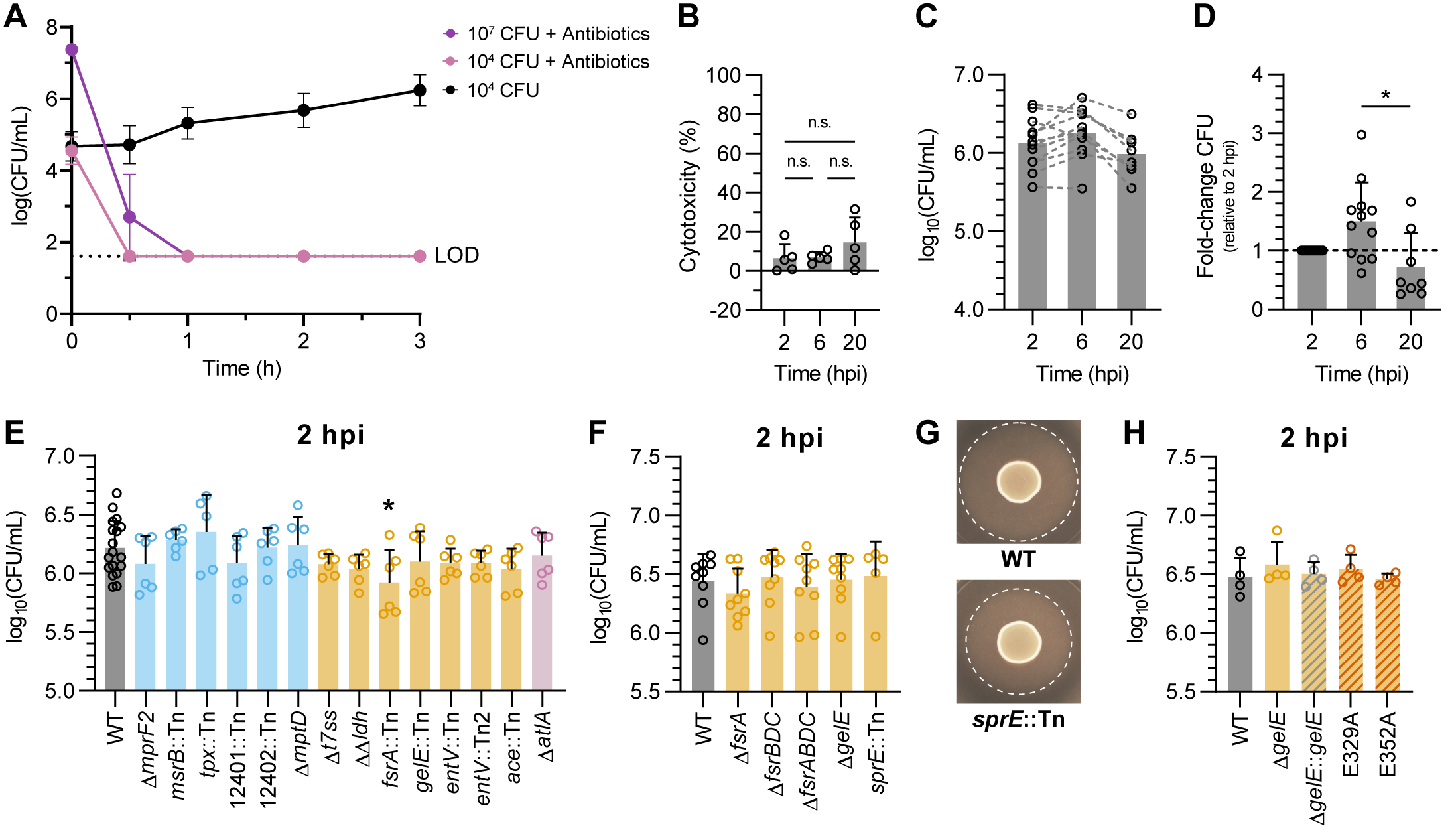

Supplement: S1 Fig — (A) Antibiotic killing kinetics under conditions used for extracellular killing of E. faecalis in the antibiotic protection assay. E. faecalis OG1RF log-phase cultures inoculated at 107 (MOI 10 equivalent; purple lines) or 104 CFU (pink lines) are not recovered after ≥ 1 h of vancomycin (10 μg/mL) + gentamicin (150 μg/mL) treatment in DMEM + 10% FBS, in the absence of host cells. Data points are mean ± SD of n = 3–4. LOD = limit of detection. (B) Cytotoxicity measurements of infected macrophages during the antibiotic protection assay. WT OG1RF infection data, shown here for ease of presentation, are also shown in Fig 5A in the experimental context (comparison to mutant strains) they were originally collected from. Statistical significance between timepoints was assessed by one-way ANOVA with Tukey’s multiple comparisons test (n = 5). n.s. = not significant. (C) Intracellular CFU in RAW264.7 macrophages infected with WT OG1RF at 2, 6, and 20 hpi using the antibiotic protection assay. Counts from the same biological replicate are connected by dotted lines (n = 8–12). (D) Fold-change analysis of intracellular CFU quantified in (C), normalized to the intracellular CFU at 2 hpi for each biological replicate. Dotted line indicates baseline CFU at 2 hpi (fold-change CFU = 1.0). Statistical significance between 6 and 20 hpi was assessed by unpaired T-test (n = 8–12). (E-F, H) Intracellular CFU in RAW264.7 macrophages infected with OG1RF-derived (E) mutants of genes implicated in intracellular persistence (blue), virulence (orange), or GelE proteolytic targets (pink) (n = 5–18), (F) genetic deletion mutants of the fsr operon and gelE as well as a transposon insertion mutant of sprE (n = 5–9) or (H) gelE-complemented strains at 2 hpi (n = 4). Statistical significance of each strain against WT was assessed using one-way ANOVA with Dunnett’s multiple comparisons test. * = p < 0.05. (G) Gelatinase activity of sprE::Tn (representative of n = 3) compared to WT (n = 1) on Todd-Hewi [file ppat.1013738.s001.tif]

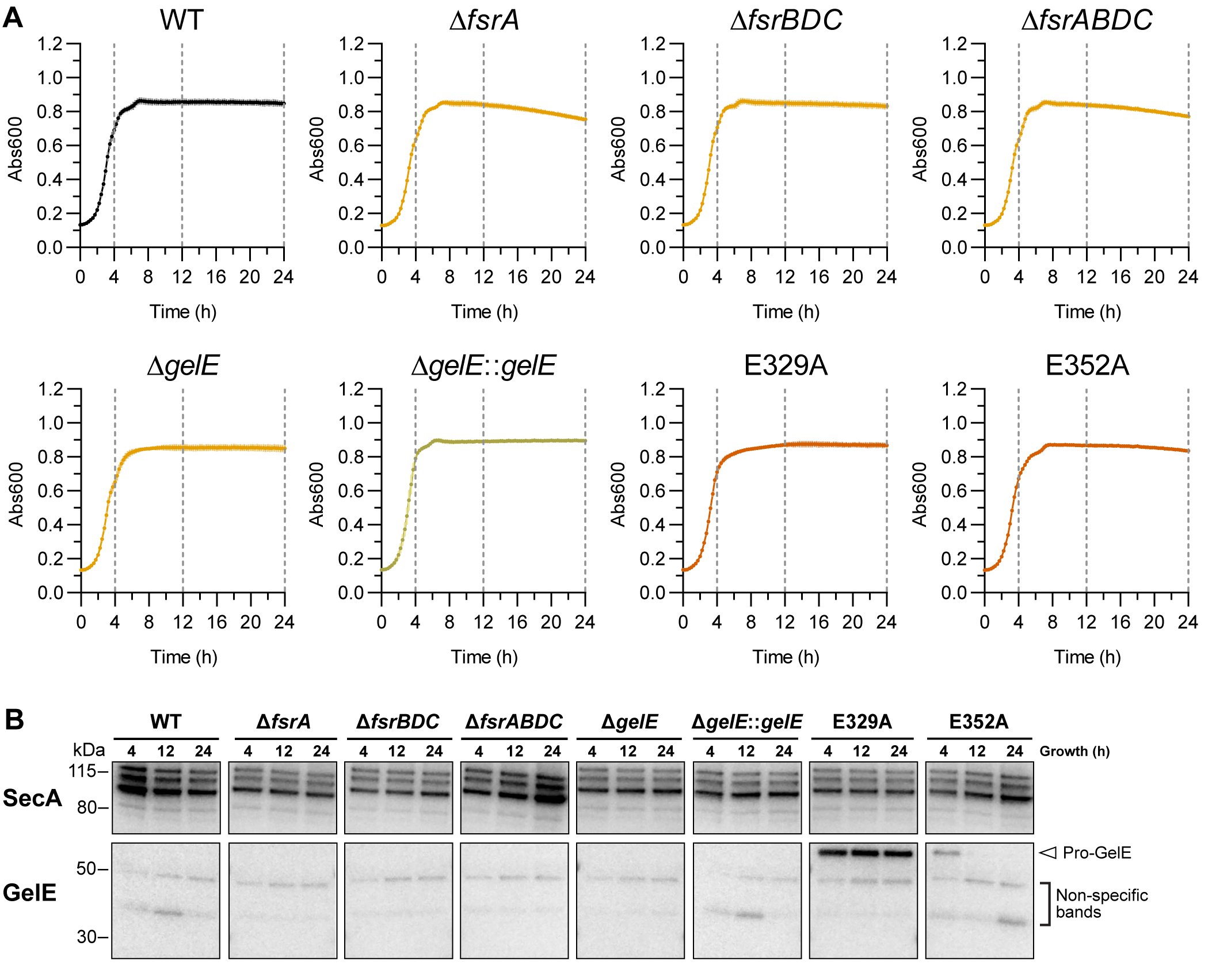

Supplement: S2 Fig — (A) Growth curve of OG1RF deletion and complementation strains in BHI broth for 24 h following 1:40 subculture from overnight cultures, plotted as mean ± SD of n = 2 with 4–5 technical replicates each. Timepoints analyzed for GelE production are marked in grey dashed lines. (B) Detection of intracellular GelE from E. faecalis cell lysates at 4, 12 and 24 h (harvested together with supernatants in Fig 1G and 1J). The membrane protein SecA was included as loading controls. White arrowheads = Pro-GelE (~55 kDa). < 50 kDa bands are also observed in ΔgelE cell lysates and are therefore likely non-specific. Images from n = 1 are shown. (TIF) [file ppat.1013738.s002.tif]

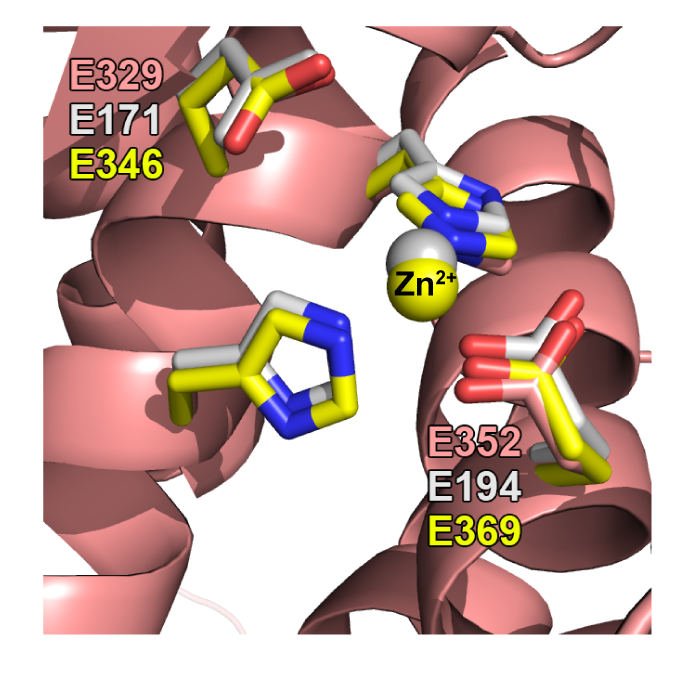

Supplement: S3 Fig — Identification of putative key GelE active site residues E329 and E352 for proteolytic activity, based on structural homology of AlphaFold2-predicted GelE structure (pink) to other M4 family zinc metalloproteases ProA (PDB 6YA1; white) and vibriolysin MCP-02 (PDB 3NQX; yellow). Based on previous studies, only mutations in residues E346 and E369 of MCP-02 (homologous to E329 and E352 of GelE respectively) produced stable, non-proteolytic proteases (Gao et al, 2010). Protein structures were aligned in WinCoot v1.1.18 using the Secondary Structure Matching (SSM) Superpose function and visualized in PyMOL v2.5.3. (TIF) [file ppat.1013738.s003.tif]

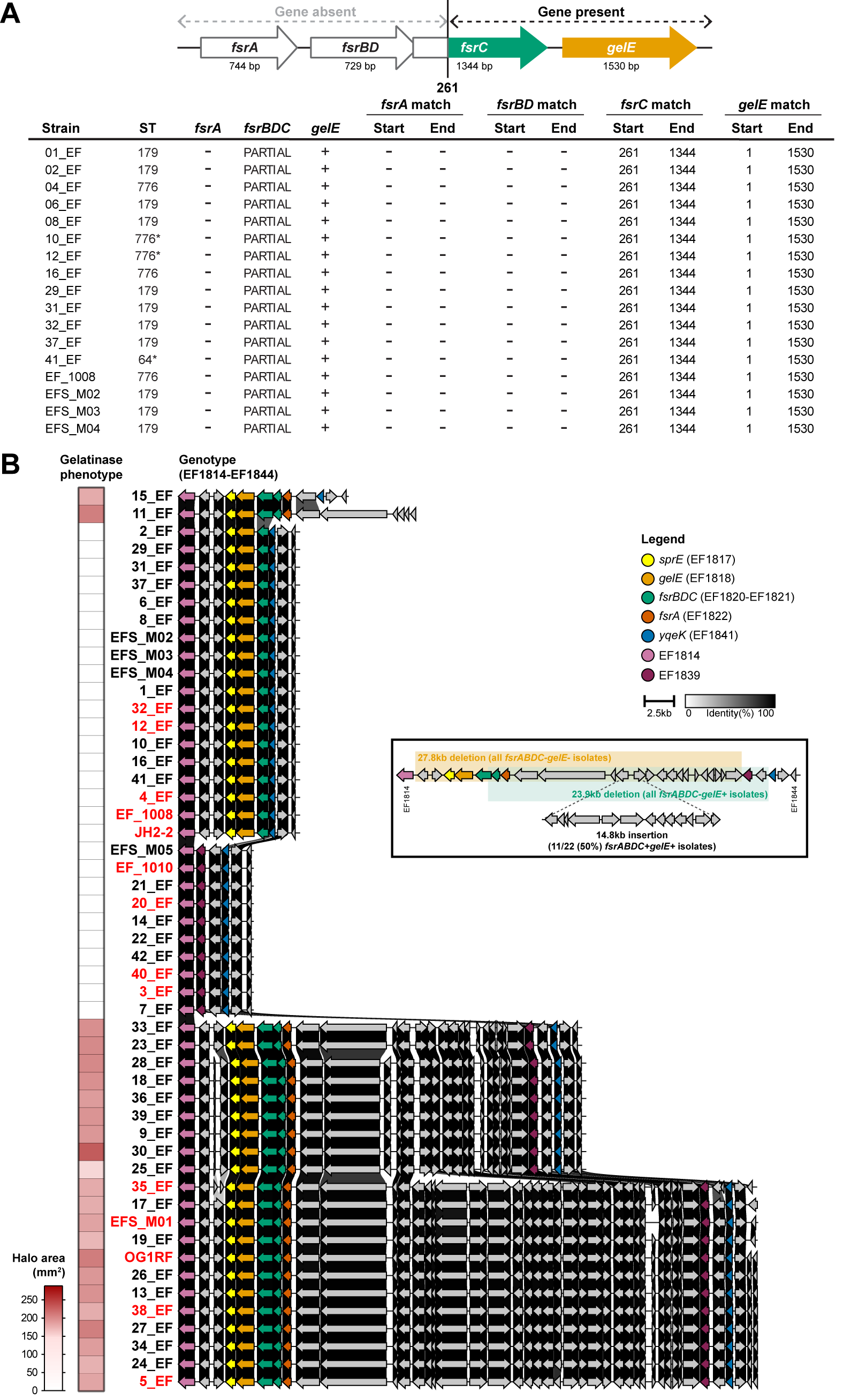

Supplement: S4 Fig — (A) Blastn query matches of contigs from all fsrABDC-gelE+ strains to fsrA, fsrBDC, fsrC or gelE. The “Start” and “End” columns indicate nucleotide positions of matches in the respective genes, while dashes indicate no matches, as visually represented by the schematic diagram. Sequence type (ST) of each strain is shown, with asterisks (*) indicating uncertainties in SRST2 sequence type calling. (B) Alignment of the genomic region corresponding to EF1814-EF1844 of the V583 reference genome for E. faecalis wound isolates. OG1RF and JH2–2 were included as reference strains. Genes with sequence identity > 30% are connected by shaded lines, colored by identity (%) as indicated in the legend. Colored arrows indicate fsrA, fsrBDC, gelE and sprE operons, as well as other genes flanking genomic deletion regions. Inset shows a diagrammatic summary of the conserved genomic deletions or insertions observed. The corresponding GelE producing phenotype for each strain (as reported in Fig 2A) are shown on the left. Strains highlighted in red are further tested by in vitro antibiotic protection assay (Fig 2B and 2D and S5). (TIF) [file ppat.1013738.s004.tif]

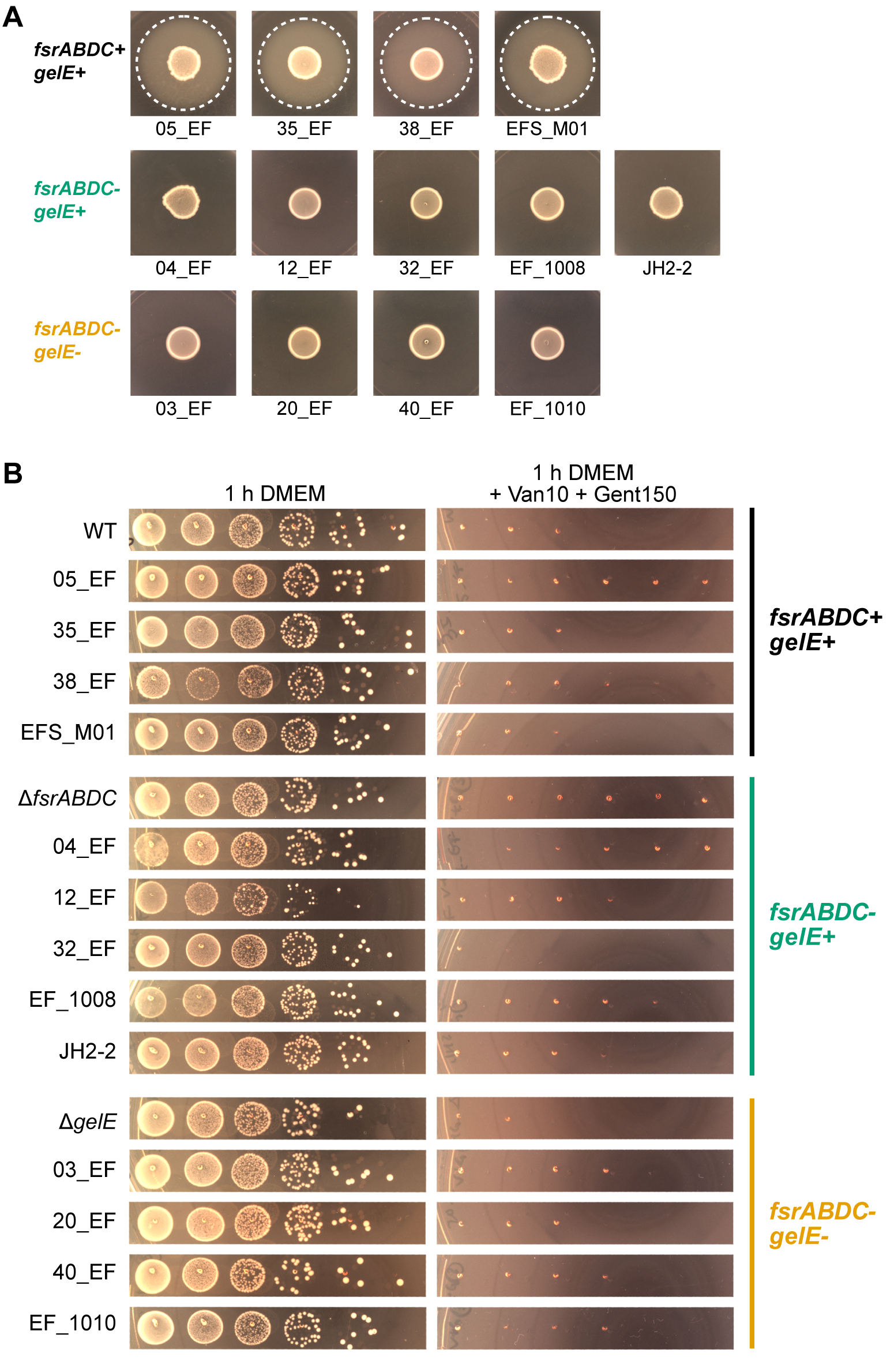

Supplement: S5 Fig — (A) Gelatinase activity of E. faecalis wound isolates and JH2–2 on Todd-Hewitt agar + 3% gelatin at 24 h. Halo formation (white dashed line) indicates gelatinase activity. Genotypes of wound isolates are indicated on the left. Representative images of n = 3 are shown. (B) Validation of bactericidal activity of vancomycin (10 μg/mL) + gentamicin (150 μg/mL) on log-phase cultures of E. faecalis wound isolates and laboratory strains (JH2–2; OG1RF WT, ΔfsrABDC, ΔgelE). Representative images from post-treatment serial dilutions are shown (n = 3). Left column = untreated controls. (TIF) [file ppat.1013738.s005.tif]

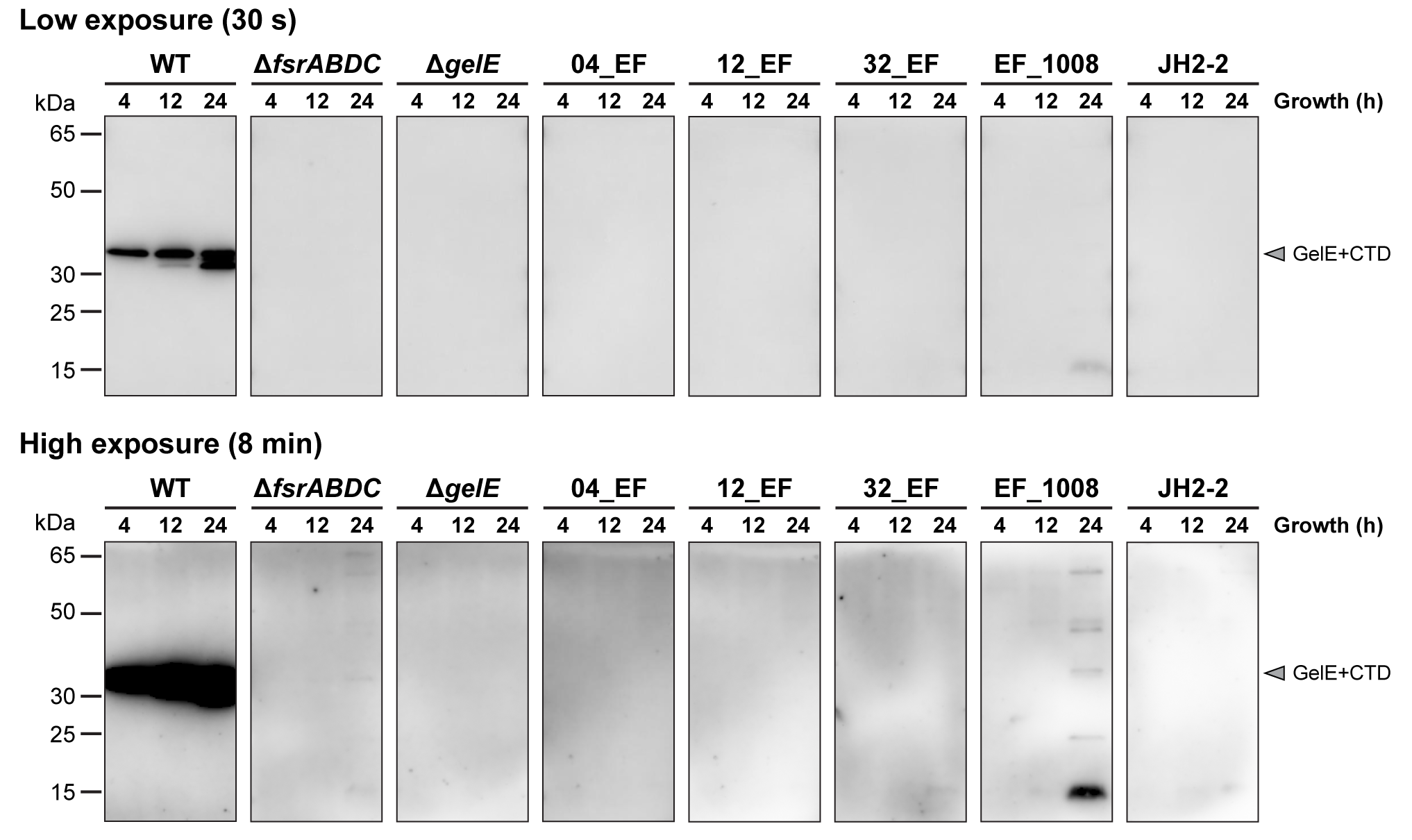

Supplement: S6 Fig — Detection of GelE secretion in culture supernatants of WT (fsrABDC+gelE+ control), ΔfsrABDC (fsrABDC-gelE+ control), ΔgelE (gelE- control), and selected clinical fsrABDC-gelE+ strains (shown in Figs 2 and S5) at 4, 12 and 24 h by Western blotting. The same blot was imaged twice at low exposure time (30 s) and high exposure time (8 min). Images from n = 1 are shown. CTD = C-terminal domain. (TIF) [file ppat.1013738.s006.tif]

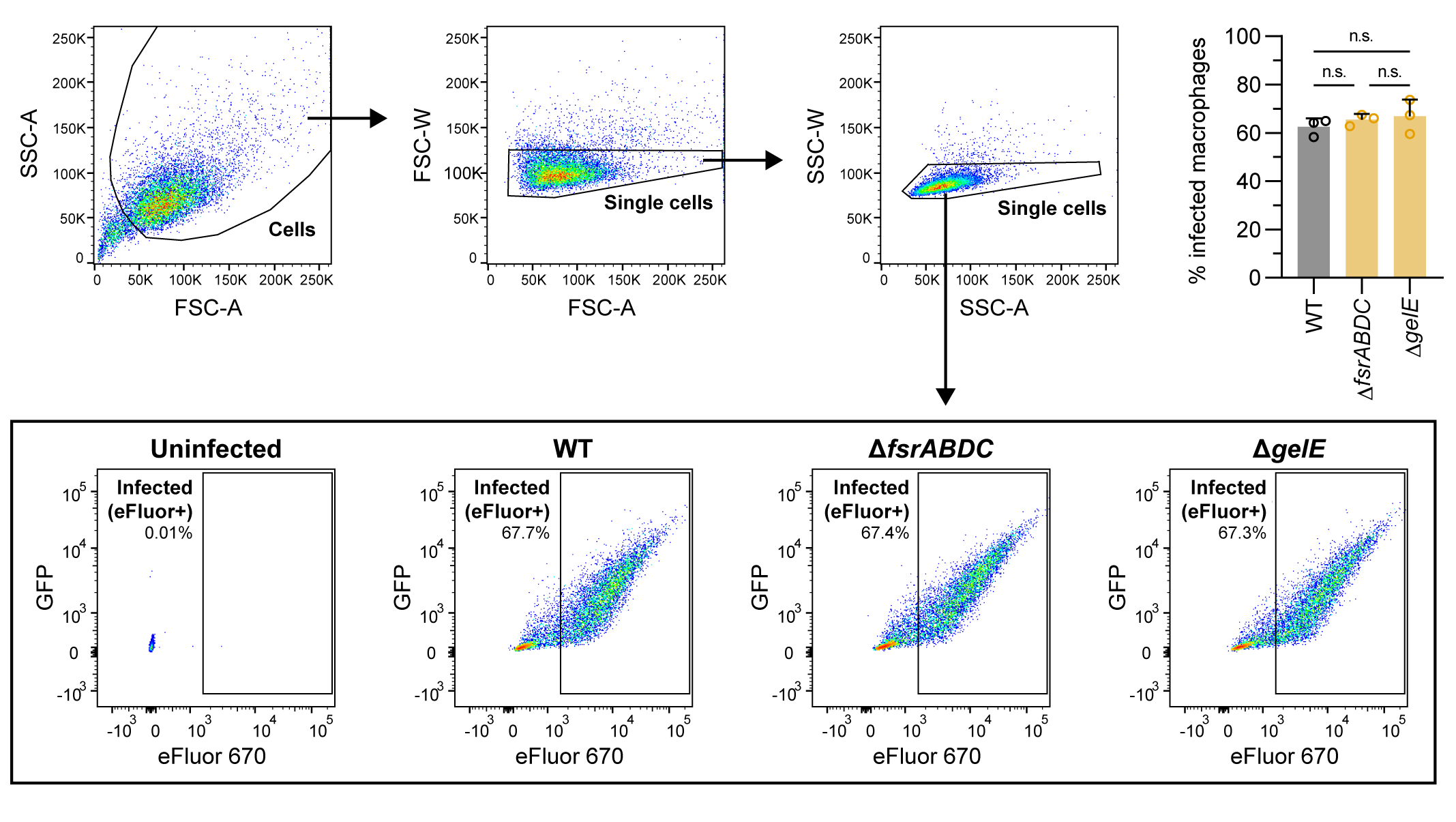

Supplement: S7 Fig — Flow cytometry analysis of RAW264.7 macrophages at 2 hpi infected with GFP-expressing WT (GelE+, gray bar), ΔfsrABDC (GelE-, orange bar) or ΔgelE (GelE-, orange bar) pre-stained with eFluor 670, showing the gating strategy and proportion of eFluor 670+ macrophages (infected macrophages). Uninfected macrophages are used as gating controls. Bars represent mean ± SD of n = 3, and representative dotplots from n = 3 are shown. Statistical significance was assessed by one-way ANOVA with Tukey’s multiple comparisons test. n.s. = not significant. (TIF) [file ppat.1013738.s007.tif]

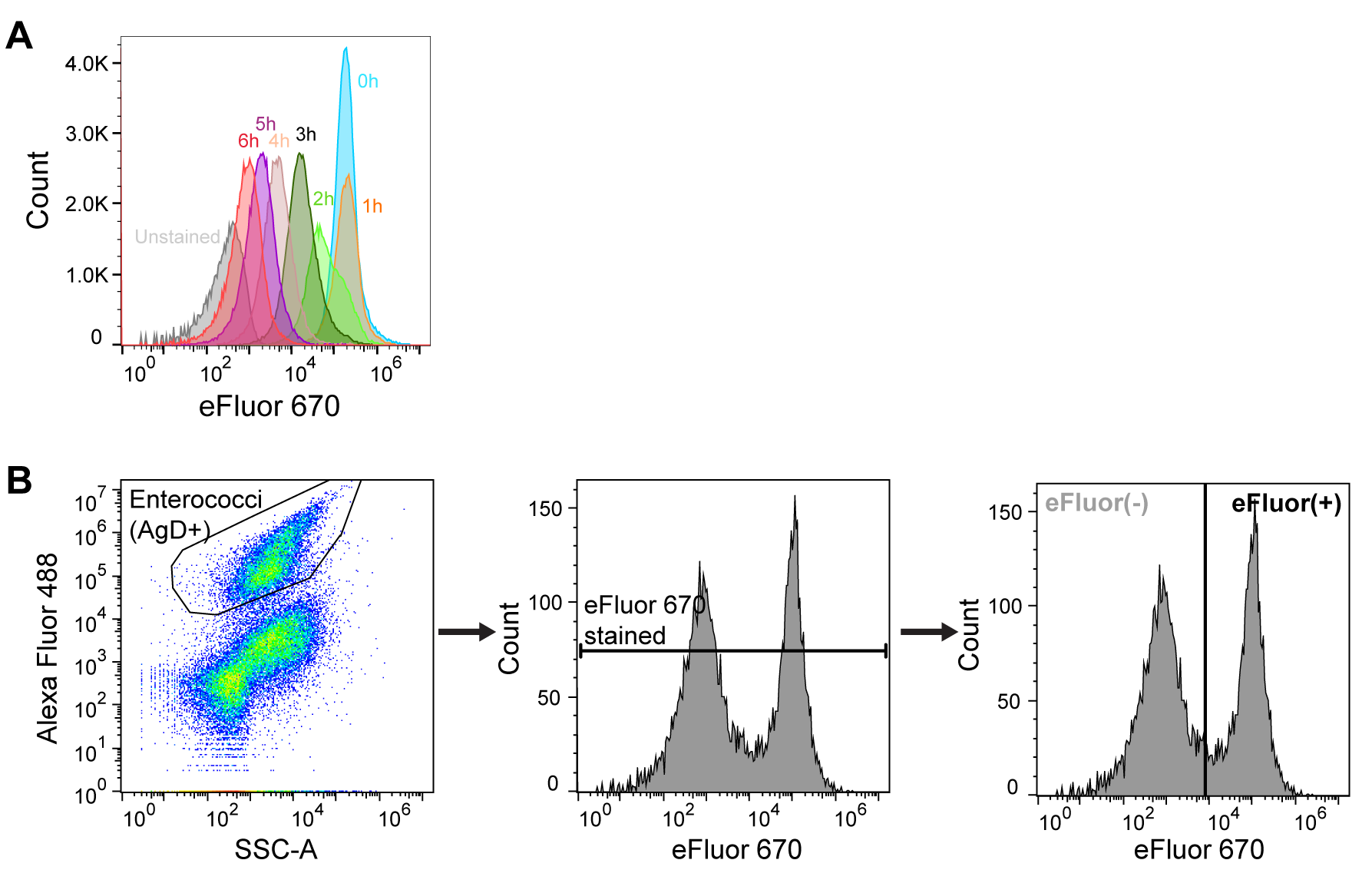

Supplement: S8 Fig — (A) Serial dilution of eFluor 670 proliferation dye in actively replicating E. faecalis pDasherGFP OG1RF cultures in colorless DMEM + 10% FBS over 6 h. Bacterial cultures were aliquoted hourly for flow cytometry analysis. Representative histograms of n = 3 are shown. (B) Gating strategy for eFluor 670 analysis of intracellular E. faecalis released from infected RAW264.7 lysates. E. faecalis stained for Group D antigen (AgD) was gated as Alexa Fluor 488+ events. Debris at the histogram edges (<100 and >107 fluorescence intensity) were gated out prior to histogram bisection for accurate quantification of eFluor+ and eFluor- events. (TIF) [file ppat.1013738.s008.tif]

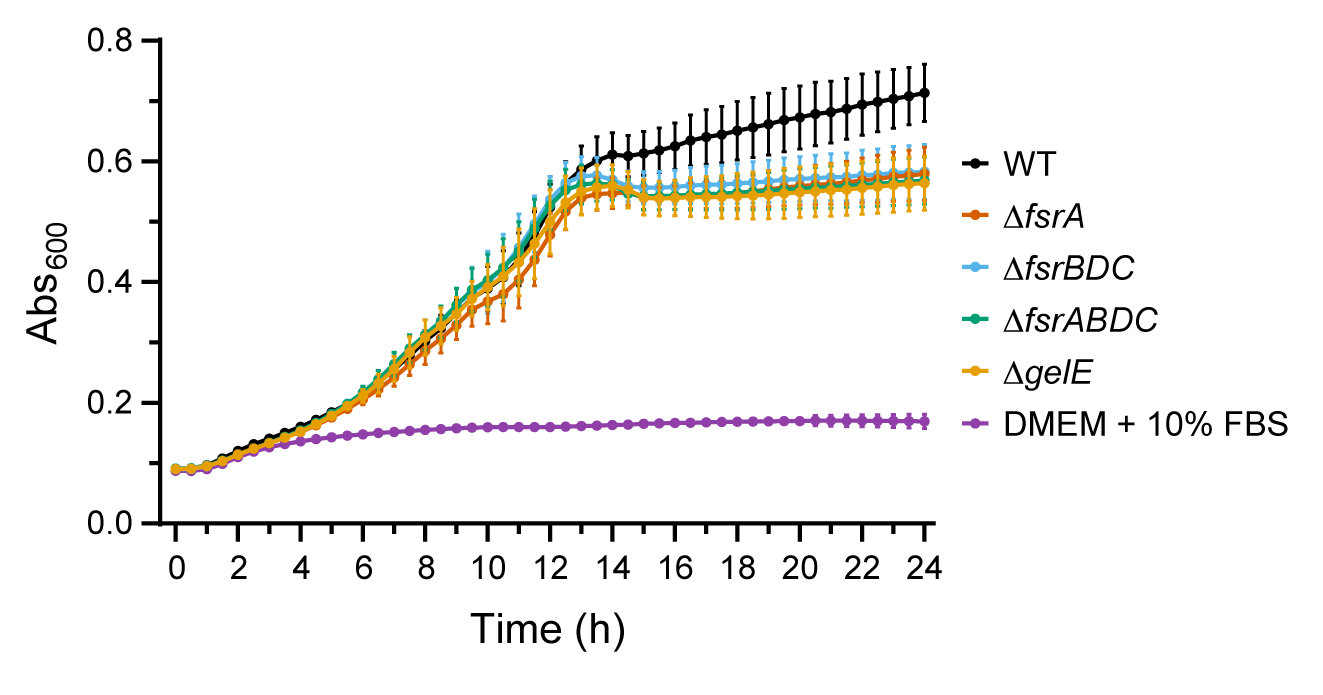

Supplement: S9 Fig — fsr/gelE deletion mutants were grown in colorless DMEM + 10% FBS for 24 h and the absorbance at 600 nm (Abs600) was measured at 30 min intervals. Colorless DMEM + 10% FBS is used as a negative control. Growth curve is plotted as a mean ± SD of n = 2 with 10 technical replicates each. (TIF) [file ppat.1013738.s009.tif]

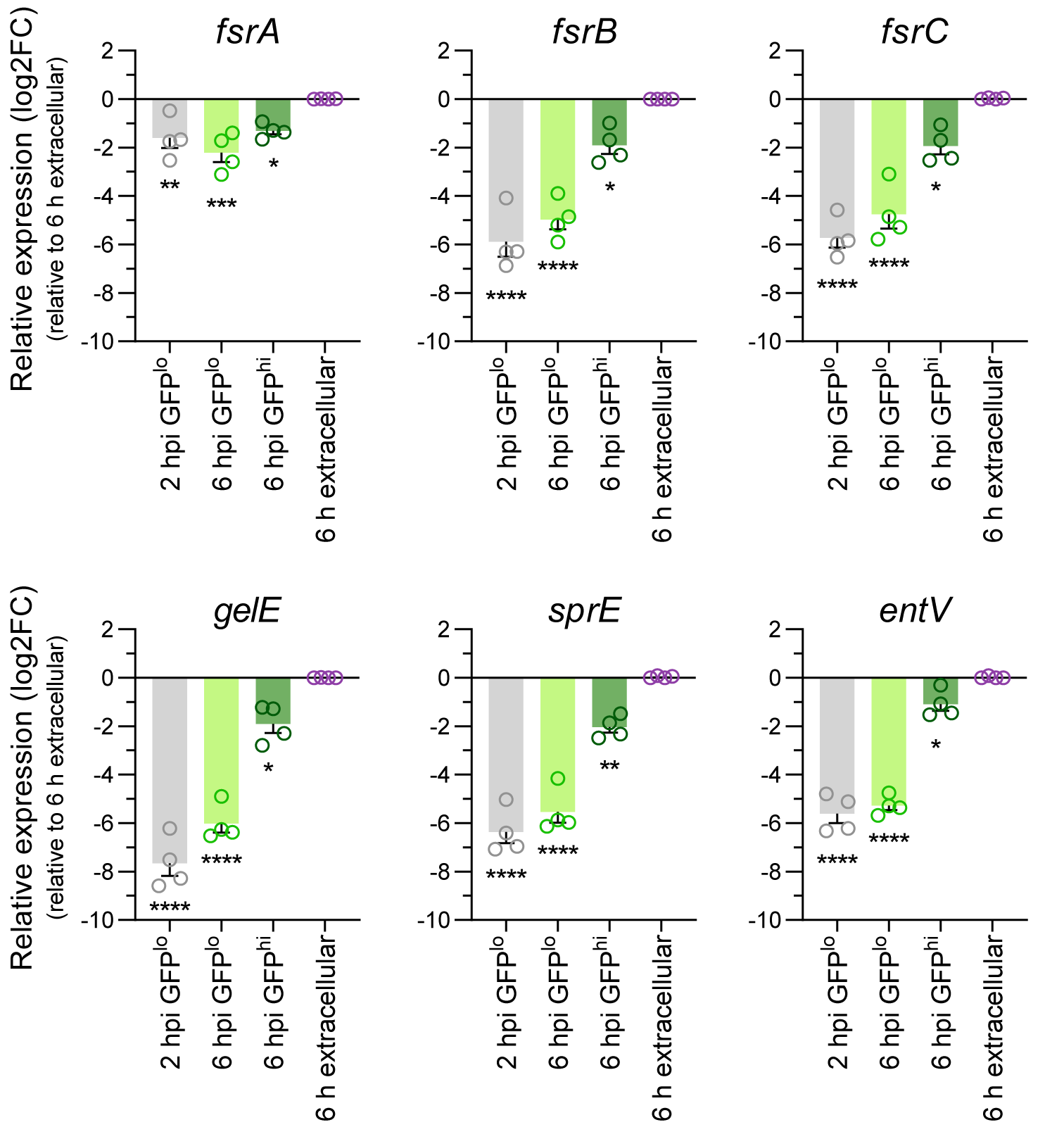

Supplement: S10 Fig — Relative gene expression of fsrABDC and its associated regulon in intracellular E. faecalis populations (2 hpi GFPlo, 6 hpi GFPlo, 6 hpi GFPhi) compared to extracellular E. faecalis grown for 6 h in the absence of macrophages. Relative expression is calculated by the -ΔΔCt method, normalised to the housekeeping gene recA and to the gene expression of 6 h extracellular E. faecalis. Bars represent mean ± SEM of n = 4. Statistical significance of each population against 6 h extracellular E. faecalis was assessed using one-way ANOVA with Dunnett’s multiple comparisons test. Only comparisons with p < 0.05 are annotated. * = p < 0.05, ** = p < 0.01, *** = p < 0.001, **** = p < 0.0001. (TIF) [file ppat.1013738.s010.tif]

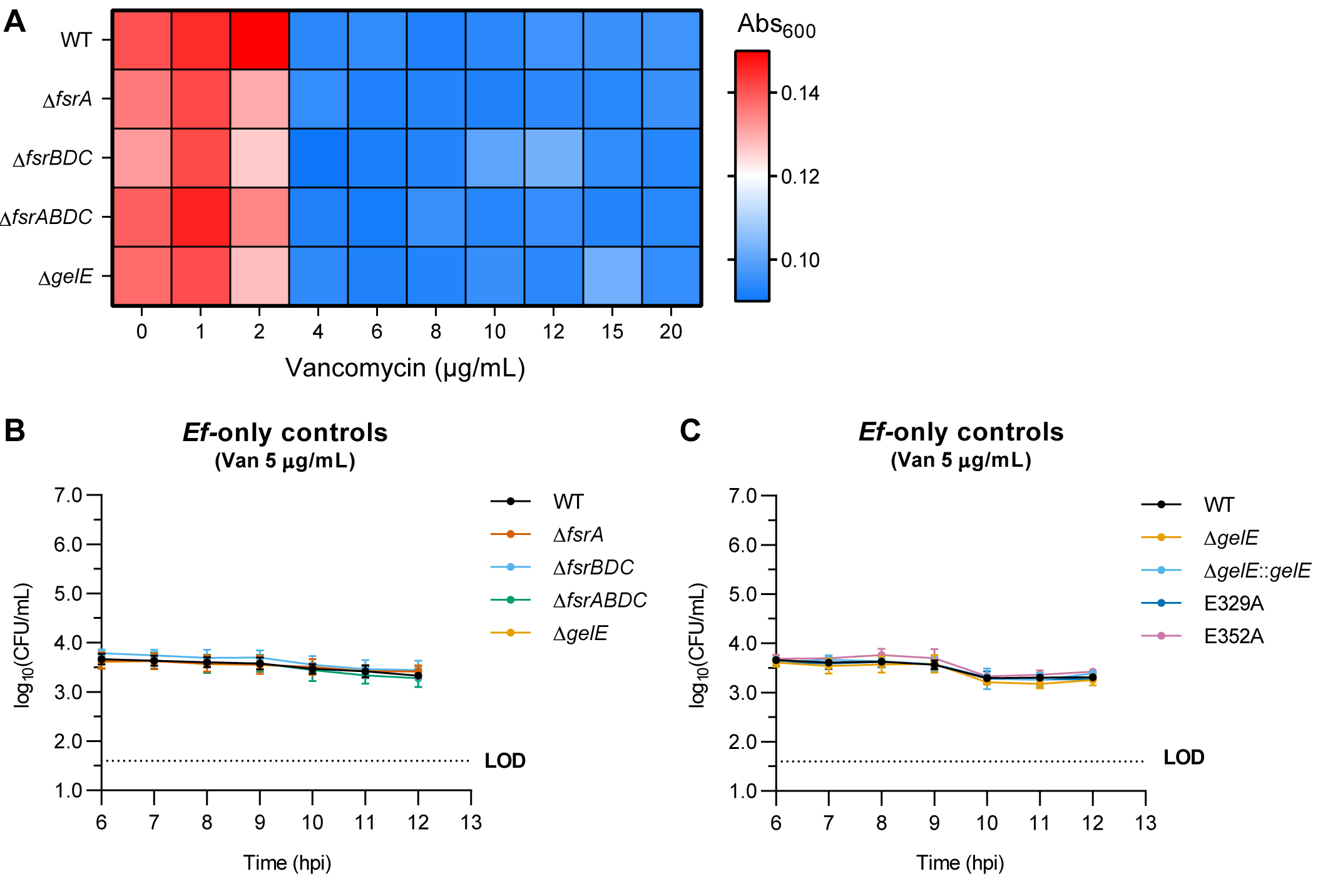

Supplement: S11 Fig — 5 μg/mL vancomycin is sufficient for a bacteriostatic effect on extracellular E. faecalis with minimal bactericidal effect. (A) Determination of minimum inhibitory concentration of vancomycin by broth microdilution in colorless DMEM + 10% FBS for OG1RF WT and fsr/gelE deletion mutants. Bacterial growth was measured as absorbance at 600 nm (Abs600), represented in the heatmap as mean of n = 2. (B-C) Validation of bacteriostatic effect of 5 μg/mL vancomycin for (B) fsr/gelE deletion mutants (n = 4) or (C) gelE-complemented strains (n = 3). Recovered bacteria CFU is expressed in log10(CFU/mL) as mean ± SD of n = 3–4. (TIF) [file ppat.1013738.s011.tif]

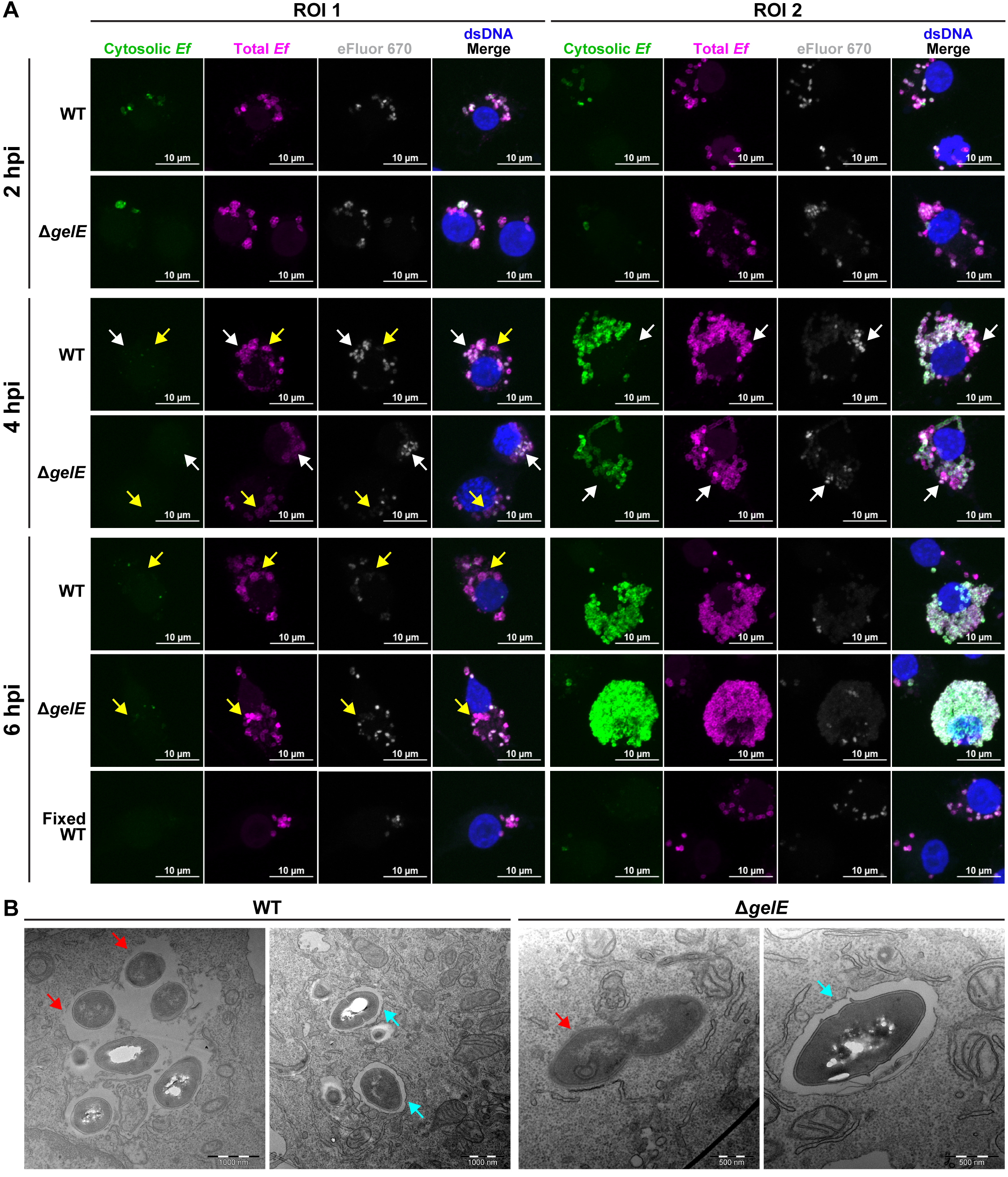

Supplement: S12 Fig — (A) Representative confocal microscopy images of RAW264.7 macrophages infected with eFluor-stained (white) WT and ΔgelE E. faecalis at 2, 4 and 6 hpi (n = 2). Samples were first weakly permeabilized by digitonin to allow staining of only cytosolic bacteria using Enterococcus-specific antibody (green), and then fully permeabilized, including permeabilization of intracellular compartments, for total bacteria staining by the same antibody (magenta), following by counterstaining for dsDNA (blue). 6 hpi infection with PFA-fixed WT E. faecalis was used as non-proliferative, non-cytosolic controls. White and yellow arrows indicate non-cytosolic bacteria population that is non-replicating (eFluor 670+) and replicating (eFluor 670-) respectively. (B) Transmission electron microscopy images of intracellular WT and ΔgelE at 6 hpi, showing non-cytosolic bacteria encapsulated by a single membrane (blue arrows) and likely cytosolic bacteria not encapsulated in a membrane-bound compartment (red arrows). Images from n = 1 are shown. (TIF) [file ppat.1013738.s012.tif]

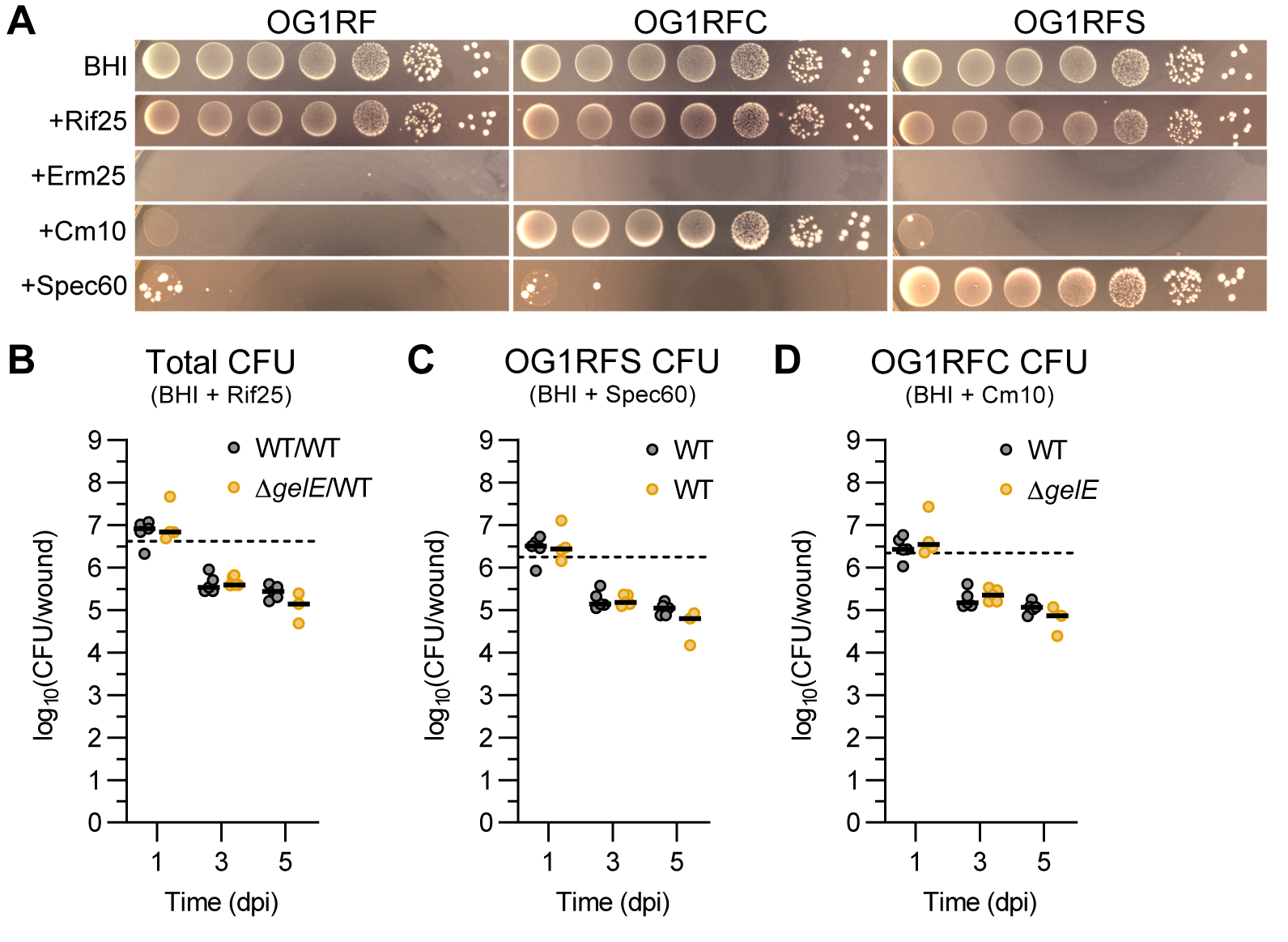

Supplement: S13 Fig — (A) Validation of growth selection of isogenic strains OG1RFC and OG1RFS on chloramphenicol (Cm, 10 μg/mL) and spectinomycin (Spec, 60 μg/mL) BHI plates respectively, compared to parental strain OG1RF. Rifampicin (Rif, 25 μg/mL) and erythromycin (Erm, 25 μg/mL) were used as positive and negative growth controls of antibiotic selection respectively. (B-D) CFU quantification from wound homogenates on antibiotic agars, selecting for (B) total OG1RFC + OG1RFS, (C) OG1RFC, and (D) OG1RFS. Black bars represent median of 3–5 animals per infection group from one independent experiment. Statistical significance between infection groups of the same timepoint was assessed using Mann-Whitney test. Only comparisons with p < 0.05 are annotated. Dotted lines show bacteria inoculum CFU. (TIF) [file ppat.1013738.s013.tif]

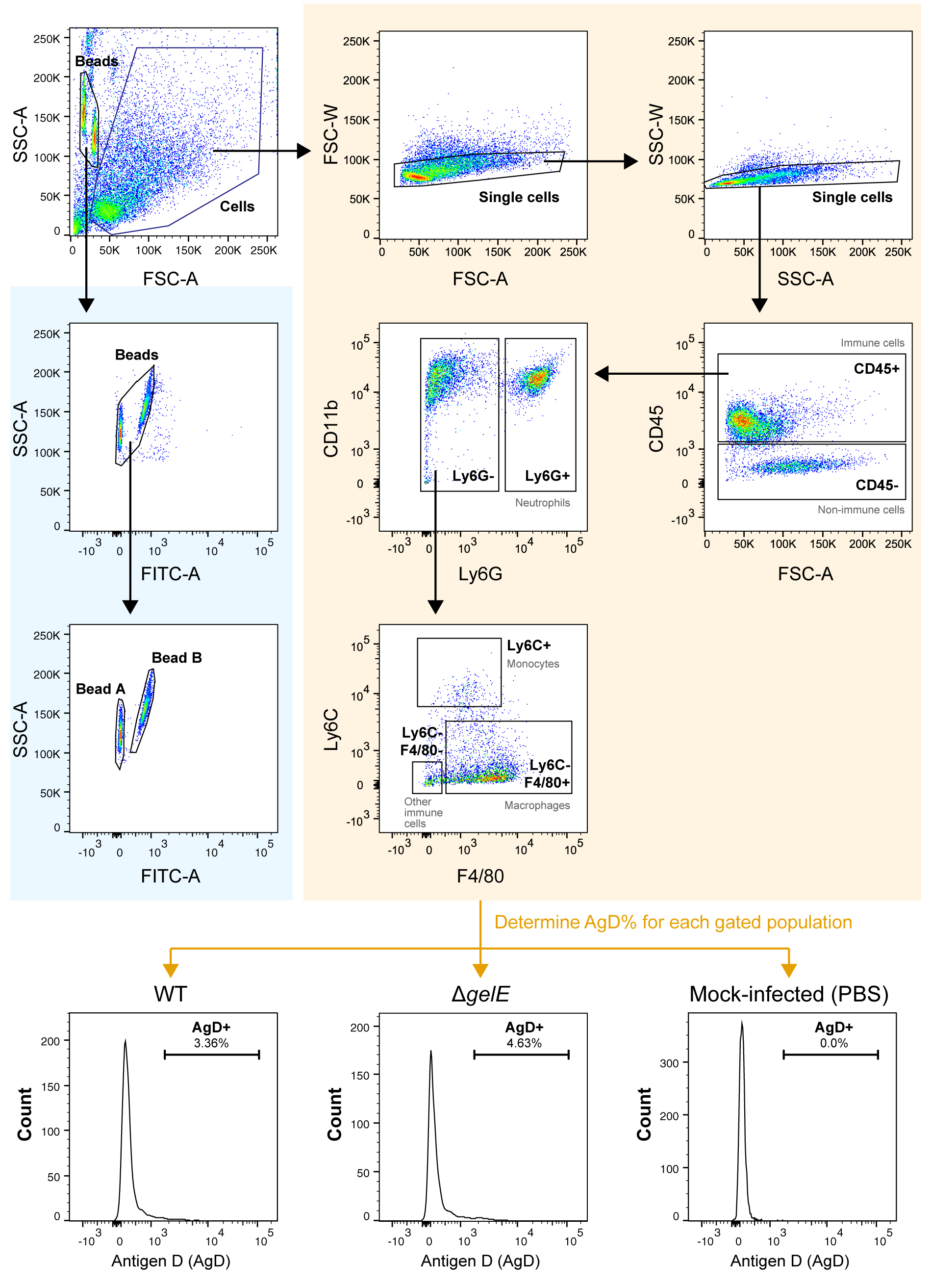

Supplement: S14 Fig — Wound cells (yellow panel) as well as AccuCheck Counting Beads A and Beads B (blue panel) were gated from forward-scatter/side-scatter (FSC/SSC) dotplots. Single cells from the wound were then separately gated for CD45+ (immune cells) or CD45- (non-immune cells), and CD45+ cells were further separated into Ly6G+ (neutrophils) and Ly6G- populations. CD45+ Ly6G- populations were subsequently gated into Ly6C+ (monocytes), Ly6C- F4/80+ (macrophages), and Ly6C- F4/80- (other immune cells) populations. Cell counts from each gated population were normalized to the total Bead A + Bead B count from each sample. Each immune cell subpopulation was further analyzed into AgD+ (infected) and AgD- (uninfected) cells, with AgD+ threshold determined in histograms based on AgD- mock-infected samples. (TIF) [file ppat.1013738.s014.tif]

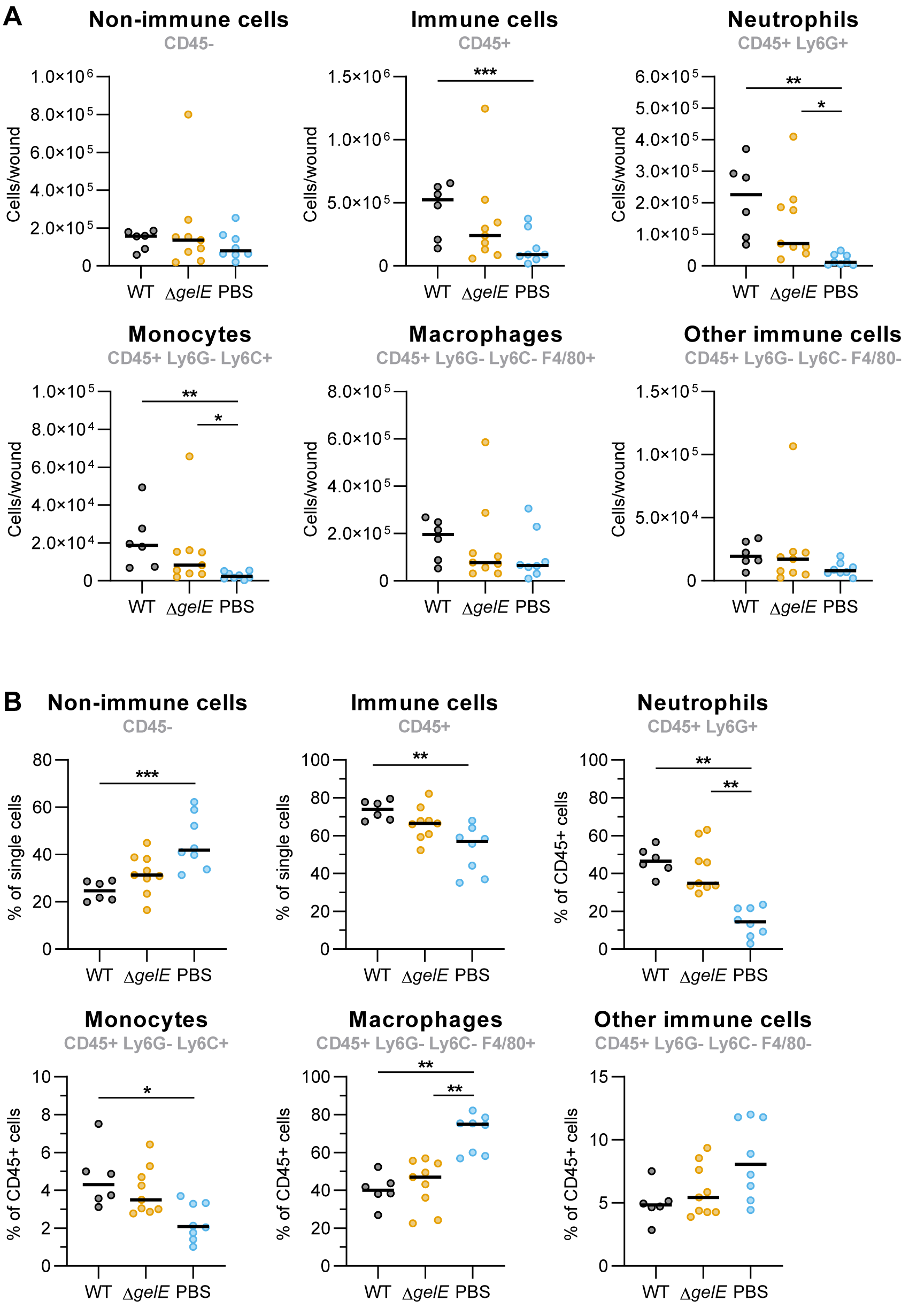

Supplement: S15 Fig — (A) Cell counts from gated populations described in S14 Fig, normalized to AccuCheck Counting Bead counts. (B) Percentage of gated populations relative to all single cells (CD45+/CD45- population) or all CD45+ cells (immune cell populations). Bars represent median from 6-9 mice from two independent experiments. For each cell population, statistical significance was assessed using Kruskal-Wallis test with Dunn’s multiple comparison test. * = p < 0.05, ** = p < 0.01, *** = p < 0.001. (TIF) [file ppat.1013738.s015.tif]
