# Supplementary material for: Gelatinase regulates the egress of intracellular replicating populations during Enterococcus faecalis infection
Source: PLoS Pathog. 2026 Mar 10;22(3):e1013738. doi: 10.1371/journal.ppat.1013738 (PMC12994788; doi:10.1371/journal.ppat.1013738)
Supplement: S1 Table — Numbers after the gene name indicate position (in bp) within the protein coding sequence where the transposon is inserted for gene disruption. (DOCX) [file ppat.1013738.s016.docx]

S1 Table. Strains used in this study. Numbers after the gene name indicate position (in bp) within the protein coding sequence where the transposon is inserted for gene disruption.

| Strain name | Description | Antibiotic resistance* | Source |
| --- | --- | --- | --- |
| OG1RF; Wildtype (WT) | Plasmid-free, gelatinase-positive strain with spontaneous Rif and Fus mutations | Rif, Fus | (1) |
| OG1RFC | OG1RF WT with a chromosomal insertion of *cat* (chloramphenicol resistance cassette) at the genomic insertion site for expression (GISE) | Rif, Fus, Cm | This study |
| OG1RFS | OG1RF WT with a chromosomal insertion of *spc* (spectinomycin resistance cassette) at the genomic insertion site for expression (GISE) | Rif, Fus, Spec | This study |
| JH2-2 | Gelatinase-negative strain with spontaneous Rif and Fus mutations | Rif, Fus | (2) |
| Δ*fsrA* | ∆OG1RF_11526; encoding response regulator FsrA | Rif, Fus | This study |
| Δ*fsrBDC* | ∆OG1RF_11527-11528; encoding membrane protein FsrB, autoinducer peptide precurse FsrD, sensor kinase FsrC | Rif, Fus | This study |
| Δ*fsrABDC* | ∆OG1RF_11526-11528; encoding FsrABDC quorum sensing TCS | Rif, Fus | (3) |
| Δ*gelE* | ∆OG1RF_11529; encoding gelatinase GelE | Rif, Fus | (4) |
| Δ*gelE*::*gelE* | Δ*gelE* with chromosomal complementation of WT *gelE* sequence with a silent A29A mutation | Rif, Fus | This study |
| E352A | Δ*gelE*::*gelE*^E352A^; Δ*gelE* with chromosomal complementation of *gelE* containing a silent A29A mutation and a zinc-coordinating E352A active site mutation | Rif, Fus | (3) |
| E329A | Δ*gelE*::*gelE*^E329A^; Δ*gelE* with chromosomal complementation of *gelE* containing a silent A29A mutation and a catalytic E329A active site mutation | Rif, Fus | This study |
| *sprE*::Tn | *sprE*666:EfaMarTn; encoding serine protease SprE | Rif, Fus, Cm | (5) |
| ∆*mprF2* | ∆OG1RF_10760; encoding multi-peptide resistance factor MprF2 | Rif, Fus | (6) |
| *msrB*::Tn | *msrB*112::EfaMarTn; encoding methionine sulfur reductase MsrB | Rif, Fus, Cm | (5) |
| 12401::Tn | OG1RF_12401(214)::EfaMarTn; encoding enzyme IID of phosphotransferase system PTS8 | Rif, Fus, Cm | (5) |
| 12402::Tn | OG1RF_12402(190)::EfaMarTn; encoding enzyme IIC of phosphotransferase system PTS8 | Rif, Fus, Cm | (5) |
| *tpx*::Tn | *tpx*349::EfaMarTn; encoding thiol peroxidase Tpx | Rif, Fus, Cm | (5) |
| ∆*mptD* | ∆OG1RF_10021; encoding enzyme IID of phosphotransferase system PTS1 (MptABCD) | Rif, Fus | (7) |
| ∆*t7ss* | ∆OG1RF_11100-OG1RF_11127; encoding the full type 7 secretion system locus | Rif, Fus | (8) |
| ∆∆*ldh* | ∆*ldh1*∆*ldh2*; ∆OG1RF_10199∆OG1RF_10373; encoding lactate dehydrogenases Ldh1 and Ldh2 | Rif, Fus | (9) |
| *fsrA::*Tn | *fsrA*10*::*EfaMarTn; encoding response regulator FsrA | Rif, Fus, Cm | (5) |
| *gelE::*Tn | *gelE*968*::*EfaMarTn; encoding gelatinase GelE | Rif, Fus, Cm | (5) |
| *entV*::Tn | *entV*(-5)::EfaMarTn; encoding enterococcin EntV | Rif, Fus, Cm | (5) |
| *entV*::Tn2 | *entV*182::EfaMarTn; encoding enterococcin EntV | Rif, Fus, Cm | (5) |
| *ace*::Tn | *ace*665::EfaMarTn; encoding collagen-binding MSCRAMM adhesin Ace | Rif, Fus, Cm | (5) |
| ∆*atlA* | ∆OG1RF_10533 (EF0799); encoding autolysin AtlA | Rif, Fus | (10) |
| OG1RFC Δ*gelE* | OG1RFC ΔOG1RF_11529; encoding gelatinase GelE | Rif, Fus | This study |

*Rif, rifampicin; Fus, fusidic acid; Cm, chloramphenicol.

**References**

1. Dunny GM, Brown BL, Clewell DB. Induced cell aggregation and mating in *Streptococcus faecalis*: evidence for a bacterial sex pheromone. Proceedings of the National Academy of Sciences. 1978;75(7):3479-83.

2. Jacob AE, Hobbs SJ. Conjugal Transfer of Plasmid-Borne Multiple Antibiotic Resistance in *Streptococcus faecalis* var. zymogenes. Journal of Bacteriology. 1974;117(2):360-72.

3. Antypas H, Schmidtchen V, Staiger WI, Yanhong LI, Tan RJW, Ng KKF, et al. Loss of Fsr quorum sensing promotes biofilm formation and worsens outcomes in enterococcal infective endocarditis. Nature Communications. 2026;17(1):1668.

4. Thomas VC, Hiromasa Y, Harms N, Thurlow L, Tomich J, Hancock LE. A fratricidal mechanism is responsible for eDNA release and contributes to biofilm development of *Enterococcus faecalis*. Molecular Microbiology. 2009;72(4):1022-36.

5. Kristich CJ, Nguyen VT, Le T, Barnes AMT, Grindle S, Dunny GM. Development and Use of an Efficient System for Random mariner Transposon Mutagenesis To Identify Novel Genetic Determinants of Biofilm Formation in the Core *Enterococcus faecalis* Genome. Applied and Environmental Microbiology. 2008;74(11):3377-86.

6. Kandaswamy K, Liew TH, Wang CY, Huston-Warren E, Meyer-Hoffert U, Hultenby K, et al. Focal targeting by human β-defensin 2 disrupts localized virulence factor assembly sites in *Enterococcus faecalis*. Proceedings of the National Academy of Sciences. 2013;110(50):20230-5.

7. Tan CAZ, Chong KKL, Yeong DYX, Ng CHM, Ismail MH, Yap ZH, et al. Purine and carbohydrate availability drive *Enterococcus faecalis* fitness during wound and urinary tract infections. mBio. 2023;15(1):e02384-23.

8. Tan AMZ, Celik C, Lee SYT, Veleba M, Manzano CS, Abdul RMK, et al. *Enterococcus faecalis* redox metabolism activates the unfolded protein response to impair wound healing. Science Advances. 2026;12(3):eaeb5297.

9. Tan CAZ, Lam LN, Biukovic G, Soh EY-C, Toh XW, Lemos JA, et al. *Enterococcus faecalis* Antagonizes *Pseudomonas aeruginosa* Growth in Mixed-Species Interactions. Journal of Bacteriology. 2022;204(7):e00615-21.

10. Guiton PS, Hung CS, Kline KA, Roth R, Kau AL, Hayes E, et al. Contribution of Autolysin and Sortase A during *Enterococcus faecalis* DNA-Dependent Biofilm Development. Infect Immun. 2009;77(9):3626-38.
