# Supplementary material for: Gelatinase regulates the egress of intracellular replicating populations during Enterococcus faecalis infection
Source: PLoS Pathog. 2026 Mar 10;22(3):e1013738. doi: 10.1371/journal.ppat.1013738 (PMC12994788; doi:10.1371/journal.ppat.1013738)
Supplement: S2 Table — Asterisks (*) indicating uncertainties in sequence type (ST) calling by SRST2. + and – indicates genotype/phenotype presence and absence, respectively. (DOCX) [file ppat.1013738.s017.docx]

**S2 Table. Details of the wound clinical isolates tested in this study.** Asterisks (*) indicating uncertainties in sequence type (ST) calling by SRST2. + and – indicates genotype/phenotype presence and absence respectively.

| Strain name | Collection site | ST | *fsrABDC* genotype | *gelE* genotype | GelE activity phenotype |
| --- | --- | --- | --- | --- | --- |
| 1_EF | Left foot | 179 | - | + | - |
| 2_EF | Perianal abscess | 179 | - | + | - |
| 3_EF | Ear cyst wound swab | 16 | - | - | - |
| 4_EF | Scrotal abscess swab | 776 | - | + | - |
| 5_EF | Right foot wound swab | 81 | + | + | + |
| 6_EF | Peripancreatic fluid | 179 | - | + | - |
| 7_EF | Wound swab | 16 | - | - | - |
| 8_EF | Left foot tissue | 179 | - | + | - |
| 9_EF | Suprapubic wound | 6 | + | + | + |
| 10_EF | Nasopharyngeal wound | 776* | - | + | - |
| 11_EF | Right lateral foot | 21 | + | + | + |
| 12_EF | Supraorbital wound | 776* | - | + | - |
| 13_EF | Buttock wound | 116 | + | + | + |
| 14_EF | Forefoot tissue | 16 | - | - | - |
| 15_EF | Left periauricular abscess | 856* | + | + | + |
| 16_EF | Wound swab | 776 | - | + | - |
| 17_EF | Sacral sore | 116 | + | + | + |
| 18_EF | Sacral swab | 6 | + | + | + |
| 19_EF | Index finger | 862 | + | + | + |
| 20_EF | Toe | 16 | - | - | - |
| 21_EF | Bile wound swab | 16 | - | - | - |
| 22_EF | Wound swab | 16 | - | - | - |
| 23_EF | Wound swab | 6 | + | + | + |
| 24_EF | Perianal abscess | 81 | + | + | + |
| 25_EF | Wound swab | 6 | + | + | + |
| 26_EF | Toe wound | 202 | + | + | + |
| 27_EF | Bile | 81 | + | + | + |
| 28_EF | Leg wound | 6 | + | + | + |
| 29_EF | Pancreatic abscess | 179 | - | + | - |
| 30_EF | Sacral swab | 6 | + | + | + |
| 31_EF | Foot swab | 179 | - | + | - |
| 32_EF | Stump | 179 | - | + | - |
| 33_EF | Wound swab | 6 | + | + | + |
| 34_EF | Heel wound swab | 81 | + | + | + |
| 35_EF | Leg wound | 314 | + | + | + |
| 36_EF | Gluteal pus | 6 | + | + | + |
| 37_EF | Peripancreatic fluid | 179 | - | + | - |
| 38_EF | Inguinal abscess | 81* | + | + | + |
| 39_EF | Tissue right forefoot | 6 | + | + | + |
| 40_EF | Wound swab | 16 | - | - | - |
| 41_EF | Tissue left lateral leg | 64* | - | + | - |
| 42_EF | Wound swab bedsore | 16 | - | - | - |
| EF_1008 | Wound swab | 776 | - | + | - |
| EF_1010 | Wound swab | 16 | - | - | - |
| EFS_M01 | Wound swab | 482 | + | + | + |
| EFS_M02 | Wound swab | 179 | - | + | - |
| EFS_M03 | Wound swab | 179 | - | + | - |
| EFS_M04 | Wound swab | 179 | - | + | - |
| EFS_M05 | Wound swab | 16 | - | - | - |
