# Supplementary material for: Gelatinase regulates the egress of intracellular replicating populations during Enterococcus faecalis infection
Source: PLoS Pathog. 2026 Mar 10;22(3):e1013738. doi: 10.1371/journal.ppat.1013738 (PMC12994788; doi:10.1371/journal.ppat.1013738)
Supplement: S3 Table — (DOCX) [file ppat.1013738.s018.docx]

S3 Table. Plasmids used in this study.

| Plasmid name | Description | Antibiotic resistance* | Source |
| --- | --- | --- | --- |
| pGCP213 | Temperature-sensitive shuttle plasmid for allelic exchange | Erm | (1) |
| pGCP213::*fsrA_del* | pGCP213::US*_fsrA_*-DS*_fsrA_* | Erm | This study |
| pGCP213::*fsrBDC_del* | pGCP213::US*_fsrBDC_*-DS*_fsrBDC_* | Erm | This study |
| pGCP213::*gelE* | pGCP213::US*_gelE_*-*gelE*(A29A)-DS*_gelE_* | Erm | This study |
| pGCP213::*gelE*^E329A^ | pGCP213::US*_gelE_*-*gelE*(A29A, E329A)-DS*_gelE_* | Erm | This study |
| pGCP213::*gelE*^E352A^ | pGCP213::US*_gelE_*-*gelE*(A29A, E352A)-DS*_gelE_* | Erm | (2) |
| pSD15 | pCR8/GW/TOPO with OG1RF_11779-MCS-OG1RF_11778 (11779-MCS-11778) for GISE constructs | Spec | (3) |
| pBSU101::DasherGFP | pBSU101 with constitutively expressed Dasher GFP under a CFB promoter (P_CFB_) | Spec | (4) |
| pSD15::DasherGFP | pSD15::11779-P_CFB_-DasherGFP-11778 | Spec | This study |
| pSD15::P*_gelE_*-DasherGFP | pSD15::11779-P*_gelE_*-DasherGFP-11778 | Spec | This study |
| pRV1 | Replicon-free shuttle plasmid containing P-*pheS** negative selection cassette | Erm | (3) |
| pFR212 | pGCP213::11779-P*_gelE_*-DasherGFP-11778 | Erm | This study |
| pFR213 | pGCP213-P-*pheS**::11779-P*_gelE_*-DasherGFP-11778 | Erm | This study |
| pFR212::*cat* | pGCP213::11779-*cat*-11778 | Erm, Cm | This study |
| pFR212::*spc* | pGCP213::11779-*spc*-11778 | Erm, Spec | This study |
| pFR213::*gelE*_del | pFR213::US*_gelE_*-DS*_gelE_* | Erm | This study |

*Erm, erythromycin; Spec, spectinomycin; Cm, chloramphenicol.

**References**

1. Nielsen HV, Guiton PS, Kline KA, Port GC, Pinkner JS, Neiers F, et al. The metal ion-dependent adhesion site motif of the *Enterococcus faecalis* EbpA pilin mediates pilus function in catheter-associated urinary tract infection. mBio. 2012;3(4):e00177-12.

2. Antypas H, Schmidtchen V, Staiger WI, Yanhong LI, Tan RJW, Ng KKF, et al. Loss of Fsr quorum sensing promotes biofilm formation and worsens outcomes in enterococcal infective endocarditis. Nature Communications. 2026;17(1):1668.

3. DebRoy S, van der Hoeven R, Singh KV, Gao P, Harvey BR, Murray BE, et al. Development of a genomic site for gene integration and expression in *Enterococcus faecalis*. Journal of Microbiological Methods. 2012;90(1):1-8.

4. Hallinen KM, Guardiola-Flores KA, Wood KB. Fluorescent reporter plasmids for single-cell and bulk-level composition assays in *E. faecalis*. PLOS ONE. 2020;15(5):e0232539.
