# Supplementary material for: Gelatinase regulates the egress of intracellular replicating populations during Enterococcus faecalis infection
Source: PLoS Pathog. 2026 Mar 10;22(3):e1013738. doi: 10.1371/journal.ppat.1013738 (PMC12994788; doi:10.1371/journal.ppat.1013738)
Supplement: S4 Table — (DOCX) [file ppat.1013738.s019.docx]

S4 Table. Primers used in this study.

| Purpose | | Primer name | Sequence | Description |  |
| --- | --- | --- | --- | --- | --- |
| Creation of Δ*fsrA* | | oFR1 | GATGCATGCTCGAGCCATCAATGATCAAACGATTTCGTA | Primer 1, for amplifying 500 bp upstream sequence of *fsrA* from WT OG1RF. 15 bp overlap for InFusion cloning is underlined. |  |
|  |  | oFR2 | CTTAATTAGTCATTATCCCTCCCTAAGTAGCCATTTGTTCACTCATCC | Primer 2, for amplifying 500 bp upstream sequence of *fsrA* from WT OG1RF and ligation to downstream sequence. Complementary to oFR3. |  |
|  |  | oFR3 | GGATGAGTGAACAAATGGCTACTTAGGGAGGGATAATGACTAATTAAG | Primer 3, for amplifying 500 bp downstream sequence of *fsrA* from WT OG1RF and ligation to upstream sequence. Complementary to oFR2. |  |
|  |  | oFR4 | TACCGAGCTCGGATCCGTTTAAATCAGATGGCTGAACAG | Primer 4, for amplifying 500 bp downstream sequence of *fsrA* from WT OG1RF. 15 bp overlap for InFusion cloning is underlined. |  |
|  |  | oFR7 | GCTCGAGCATGCATCTAGAGG | Primer F, for inverse PCR (linearisation) of pGCP213 to insert *fsrA* deletion cassette. 15 bp overlap for InFusion cloning is underlined. |  |
|  |  | oFR8 | GATCCGAGCTCGGTACCAAG | Primer R, for inverse PCR (linearisation) of pGCP213 to insert *fsrA* deletion cassette. 15 bp overlap for InFusion cloning is underlined. |  |
|  |  | M13F_ pUC (-40) | GTTTTCCCAGTCACGAC | Universal sequencing primer F for pFR213 inserts. |  |
|  |  | M13R_ pUC (-26) | GTCATAGCTGTTTCCTG | Universal sequencing primer R for pFR213 inserts. |  |
|  |  | oFR5 | TGACAAGAACAGTTTGGCGG | Sequencing primer F for *fsrA* deletion |  |
|  |  | oFR6 | AAGGTTTCGCTTAACGTCCC | Sequencing primer R for *fsrA* deletion |  |
| Creation of Δ*fsrBDC* | | oFR15 | GATGCATGCTCGAGCCAATTTATTCAAAAGAACGTGGATTTTC | Primer 1, for amplifying 502 bp upstream sequence of *fsrBDC* from WT OG1RF. 15 bp overlap for InFusion cloning is underlined. |  |
|  |  | oFR16 | CCATAGCAAAAAAGTTGTTAACAAATTCATTCCATATCGCCCTCCTCTTCAAG | Primer 2, for amplifying 502 bp upstream sequence of *fsrBDC* from WT OG1RF and ligation to downstream sequence. Complementary to oFR3. |  |
|  |  | oFR17 | CTTGAAGAGGAGGGCGATATGGAATGAATTTGTTAACAACTTTTTTGCTATGG | Primer 3, for amplifying 507 bp downstream sequence of *fsrBDC* from WT OG1RF and ligation to upstream sequence. Complementary to oFR2. |  |
|  |  | oFR18 | TACCGAGCTCGGATCCACATTATCTGAATCAACAGTAACGC | Primer 4, for amplifying 507 bp downstream sequence of *fsrBDC* from WT OG1RF. 15 bp overlap for InFusion cloning is underlined. |  |
|  |  | oFR7 | GCTCGAGCATGCATCTAGAGG | Primer F, for inverse PCR (linearisation) of pGCP213 to insert *fsrBDC* deletion cassette. 15 bp overlap for InFusion cloning is underlined. |  |
|  |  | oFR8 | GATCCGAGCTCGGTACCAAG | Primer R, for inverse PCR (linearisation) of pGCP213 to insert *fsrBDC* deletion cassette. 15 bp overlap for InFusion cloning is underlined. |  |
|  |  | M13F_ pUC (-40) | GTTTTCCCAGTCACGAC | Universal sequencing primer F for pFR213 inserts. |  |
|  |  | M13R_ pUC (-26) | GTCATAGCTGTTTCCTG | Universal sequencing primer R for pFR213 inserts. |  |
|  |  | oFR19 | ACGGAACGGAAGTACCAGTC | Sequencing primer F for *fsrBDC* deletion |  |
|  |  | oFR20 | AGCTGCCTCAGAAATTGCCT | Sequencing primer R for *fsrBDC* deletion |  |
| Creation of *gelE-*complementation strains | | oFR94 | GATGCATGCTCGAGCGAATTGAAAATGTTCGCTATCTC | Primer 1, for amplifying *gelE* from WT OG1RF (including 519 bp upstream sequence). 15 bp overlap for InFusion cloning is underlined. |  |
|  |  | oFR96 | GAATAAACTTGTTCTTC**CGC**GGC | Primer 2, mutagenic primer for A29A silent mutation. Overlaps with oFR99 (primer 3). The GCA > GCG silent mutation is in bold. |  |
|  |  | oFR99 | GTAGCC**GCG**GAAGAACAAG | Primer 3, mutagenic primer for A29A silent mutation. Overlaps with oFR96 (primer 2). The GCA > GCG silent mutation is in bold. |  |
|  |  | oFR98 | TACCGAGCTCGGATCGACGATCGTTTTGTTTGC | Primer 4, for amplifying *gelE* from WT OG1RF (including 526 bp downstream sequence). 15 bp overlap for InFusion cloning is underlined. |  |
|  |  | oFR80 | GTTGGTCAT**GCA**ATGACACATGGTG | Mutagenic primer 1 for E329A site-directed mutagenesis of *gelE*. Overlaps with oFR81. The GAA > GCA (E > A) mutation is in bold. |  |
|  |  | oFR81 | CACCATGTGTCAT**TGC**ATGACCAAC | Mutagenic primer 2 for E329A site-directed mutagenesis of *gelE*. Overlaps with oFR80. The GAA > GCA (E > A) mutation is in bold. |  |
|  |  | oFR82 | CAGGTGCCTTGAAT**GCA**TCTTATTCTG | Mutagenic primer 1 for E352A site-directed mutagenesis of *gelE*. Overlaps with oFR83. The GAA > GCA (E > A) mutation is in bold. |  |
|  |  | oFR83 | CAGAATAAGA**TGC**ATTCAAGGCACC | Mutagenic primer 2 for E352A site-directed mutagenesis of *gelE*. Overlaps with oFR82. The GAA > GCA (E > A) mutation is in bold. |  |
|  |  | M13F_ pUC (-40) | GTTTTCCCAGTCACGAC | Universal sequencing primer F for pGCP213 inserts. |  |
|  |  | M13R_ pUC (-26) | GTCATAGCTGTTTCCTG | Universal sequencing primer R for pGCP213 inserts. |  |
|  |  | oFR109 | GCAACAAATATTTACGCAGGGAAAG | Sequencing primer F for colony PCR of correct *gelE*-complemented clones. |  |
|  |  | oFR110 | GACAGGCTAAAACCGGCTATC | Sequencing primer R for colony PCR of correct *gelE*-complemented clones. |  |
| Construction of pFR212/ pFR213 | | oFR43 | | GGCGGCCGCGAGCTCAATTCATCTAAAATAGTACGCTTCT | Primer F, for amplifying P_CFB_-DasherGFP insert from pBSU101::DasherGFP. SacI restriction site is underlined. |
|  |  | oFR44 | | GGGTACCACGCATGCTTACTGATACGTGTCCAGATC | Primer R, for amplifying P_CFB_-DasherGFP insert from pBSU101::DasherGFP. SphI restriction site is underlined. |
|  |  | oFR102 | | CTATTTTAGATGAATTGAGCTCGCGG | Primer F, for inverse PCR (linearisation) of pSD15::DasherGFP for P*_gelE_* replacement. 15 bp overlap for InFusion cloning is underlined. |
|  |  | oFR103 | | ATGACGGCATTGACGGAAG | Primer F, for inverse PCR (linearisation) of pSD15::DasherGFP for P*_gelE_* replacement. 15 bp overlap for InFusion cloning is underlined. |
|  |  | oFR104 | | ATTCATCTAAAATAGGAGTTATGAGGGGCAATACAG | Primer F, for amplifying P*_gelE_* from WT OG1RF. 15 bp overlap for InFusion cloning is underlined. |
|  |  | oFR105 | | CGTCAATGCCGTCATCAAACAATTAACTCCTTCCC | Primer R, for amplifying P*_gelE_* from WT OG1RF. 15 bp overlap for InFusion cloning is underlined. |
|  |  | oFR100 | | ATGCATGCTCGAGCATGGCAACTATCACAGATATC | Primer F, for amplifying GISE cassette from pSD15::P*_gelE_*-DasherGFP. 14 bp overlap for InFusion cloning is underlined. |
|  |  | oFR101 | | GTACCGAGCTCGGATCATGACGTGTATTTCATTATCGA | Primer R, for amplifying GISE cassette from pSD15::P*_gelE_*-DasherGFP. 16 bp overlap for InFusion cloning is underlined. |
|  |  | oFR7 | | GCTCGAGCATGCATCTAGAGG | Primer F, for inverse PCR (linearisation) of pGCP213 for GISE cassette insertion. 14 bp overlap for InFusion cloning is underlined. |
|  |  | oFR8 | | GATCCGAGCTCGGTACCAAG | Primer R, for inverse PCR (linearisation) of pGCP213 for GISE cassette insertion. 16 bp overlap for InFusion cloning is underlined. Also used as sequencing primer F for P-*pheS** insertion. |
|  |  | oFR39 | | AACCTGTCGTGCCAGCTG | Primer F, for inverse PCR (linearisation) of pFR212 for P-*pheS** insertion. 15 bp overlap for InFusion cloning is underlined. |
|  |  | oFR40 | | TCCCGACTGGAAAGCGG | Primer R, for inverse PCR (linearisation) of pFR212 for P-*pheS** insertion. 15 bp overlap for InFusion cloning is underlined. |
|  |  | oFR41 | | CTGGCACGACAGGTTCTAAGCTTGATTTTCGTTCGTG | Primer F, for amplifying P-*pheS** from pRV1. 15 bp overlap for InFusion cloning is underlined. |
|  |  | oFR42 | | GCTTTCCAGTCGGGACTGTCCGCTAATTCTTGCG | Primer R, for amplifying P-*pheS** from pRV1. 15 bp overlap for InFusion cloning is underlined. |
|  |  | oFR10 | | GGAAATGATTTACCTACTGCG | Sequencing primer F for inserts between OG1RF_11778 and OG1RF_11779. |
|  |  | oFR11 | | TTGCAATGCGATTGACG | Sequencing primer R for inserts between OG1RF_11778 and OG1RF_11779. |
|  |  | M13F_ pUC (-40) | | GTTTTCCCAGTCACGAC | Universal sequencing primer F for pSD15/pGCP213 inserts. |
|  |  | M13R_ pUC (-26) | | GTCATAGCTGTTTCCTG | Universal sequencing primer R for pSD15/pGCP213 inserts. |
|  |  | oFR8 | | GATCCGAGCTCGGTACCAAG | Sequencing primer F for P-*pheS** insertion. |
|  |  | oFR14 | | CTAGTGTAGCCGTAGTTAGGC | Sequencing primer R for P-*pheS** insertion. |
| Creation of OG1RFC/ OG1RFS | | oFR102 | | CTATTTTAGATGAATTGAGCTCGCGG | Primer F, for inverse PCR (linearisation) of pFR212 for P*_gelE_*-DasherGFP replacement. 15 bp overlap for InFusion cloning is underlined. |
|  |  | oFR118 | | ATCAGTAAGCATGCGTGGTAC | Primer R, for inverse PCR (linearisation) of pFR212 for P*_gelE_*-DasherGFP replacement. 15 bp overlap for InFusion cloning is underlined. |
|  |  | oFR116 | | ATTCATCTAAAATAGGTCACTAGTAAAGCGAACGAAAAAC | Primer F, for amplifying *cat* from EfaMarTn cassette. 15 bp overlap for InFusion cloning is underlined. |
|  |  | oFR117 | | CGCATGCTTACTGATAGAATGCGTGTGCTCTGC | Primer R, for amplifying *cat* from EfaMarTn cassette. 15 bp overlap for InFusion cloning is underlined. |
|  |  | oFR127 | | ATTCATCTAAAATAGGAAAAAATCGCTATAATGACCC | Primer F, for amplifying *spc* from pBSU101::DasherGFP. 15 bp overlap for InFusion cloning is underlined. |
|  |  | oFR128 | | CGCATGCTTACTGATTTCAATAGTTACAAATTGTTTCAC | Primer R, for amplifying *spc* from pBSU101::DasherGFP. 15 bp overlap for InFusion cloning is underlined. |
|  |  | oFR10 | | GGAAATGATTTACCTACTGCG | Sequencing primer F for inserts between OG1RF_11778 and OG1RF_11779. |
|  |  | oFR11 | | TTGCAATGCGATTGACG | Sequencing primer R for inserts between OG1RF_11778 and OG1RF_11779. |
| Creation of OG1RFC Δ*gelE* | | oFR94 | | GATGCATGCTCGAGCGAATTGAAAATGTTCGCTATCTC | Primer 1, for amplifying 519 bp upstream sequence of *gelE* from WT OG1RF. 15 bp overlap for InFusion cloning is underlined. |
|  |  | oFR129 | | TCATTCATTGACCAGAACAGACTTCATCAAACAATTAACTCCT | Primer 2, for amplifying 519 bp upstream sequence of *gelE* from WT OG1RF and ligation to downstream sequence. Complementary to oFR130. |
|  |  | oFR130 | | AGGAGTTAATTGTTTGATGAAGTCTGTTCTGGTCAATGAATGA | Primer 2, for amplifying 526 bp downstream sequence of *gelE* from WT OG1RF and ligation to upstream sequence. Complementary to oFR129. |
|  |  | oFR98 | | TACCGAGCTCGGATCGACGATCGTTTTGTTTGC | Primer 1, for amplifying 526 bp downstream sequence of *gelE* from WT OG1RF. 15 bp overlap for InFusion cloning is underlined. |
|  |  | oFR7 | | GCTCGAGCATGCATCTAGAGG | Primer F, for inverse PCR (linearisation) of pGCP213 to insert *gelE* deletion cassette. 15 bp overlap for InFusion cloning is underlined. |
|  |  | oFR8 | | GATCCGAGCTCGGTACCAAG | Primer R, for inverse PCR (linearisation) of pGCP213 to insert *gelE* deletion cassette. 15 bp overlap for InFusion cloning is underlined. |
|  |  | M13F_ pUC (-40) | | GTTTTCCCAGTCACGAC | Universal sequencing primer F for pFR213 inserts. |
|  |  | M13R_ pUC (-26) | | GTCATAGCTGTTTCCTG | Universal sequencing primer R for pFR213 inserts. |
|  |  | oFR109 | | GCAACAAATATTTACGCAGGGAAAG | Sequencing primer F for colony PCR of correct *gelE* deletion clones. |
|  |  | oFR110 | | GACAGGCTAAAACCGGCTATC | Sequencing primer R for colony PCR of correct *gelE* deletion clones. |
| Quantitative PCR (qPCR) | | fsrA_F | | AGCAACCTCAAATCCTGCCT | qPCR primer F for *fsrA* |
|  |  | fsrA_R | | TACAAGTGGCACACCAGGAC | qPCR primer R for *fsrA* |
|  |  | fsrB_F | | AGACCTTGGATGACGAGACCG | qPCR primer F for *fsrB* |
|  |  | fsrB_R | | GGTATGCGCCACAAGGAACA | qPCR primer R for *fsrB* |
|  |  | fsrC_F | | TGCACTGTTTTCAATCGCGT | qPCR primer F for *fsrC* |
|  |  | fsrC_R | | ACCGCAAAGCAAGCAAAACT | qPCR primer R for *fsrC* |
|  |  | gelE_F | | ACAAGATGGGCATCCCTCGA | qPCR primer F for *gelE* |
|  |  | gelE_R | | TCAAGCGCCATCACTAGCGA | qPCR primer R for *gelE* |
|  |  | sprE_F | | ATTGCGGTAGTGACTGTCGG | qPCR primer F for *sprE* |
|  |  | sprE_R | | CGACCATTGCGTGTGGTTTT | qPCR primer R for *sprE* |
|  |  | entV_F | | AGCTGCACAAAAGAAAGCCTG | qPCR primer F for *entV* |
|  |  | entV_R | | TAGCCCACATTGAACTGCCC | qPCR primer R for *entV* |
|  |  | recA_F | | GCGGCTGTTCCACCATTTCG | qPCR primer F for *recA* (housekeeping gene) |
|  |  | recA_R | | GTTGCATTGGGCGTAGGTGG | qPCR primer R for *recA* (housekeeping gene) |
