# Supplementary material for: Gelatinase regulates the egress of intracellular replicating populations during Enterococcus faecalis infection
Source: PLoS Pathog. 2026 Mar 10;22(3):e1013738. doi: 10.1371/journal.ppat.1013738 (PMC12994788; doi:10.1371/journal.ppat.1013738)
Supplement: S1 Data — (PDF) [file ppat.1013738.s020.pdf]

Fig 1G, 1J. Absence of *fsrA* and *gelE* enhances intracellular survival of *E. faecalis* in RAW264.7 macrophages at 20 hpi.

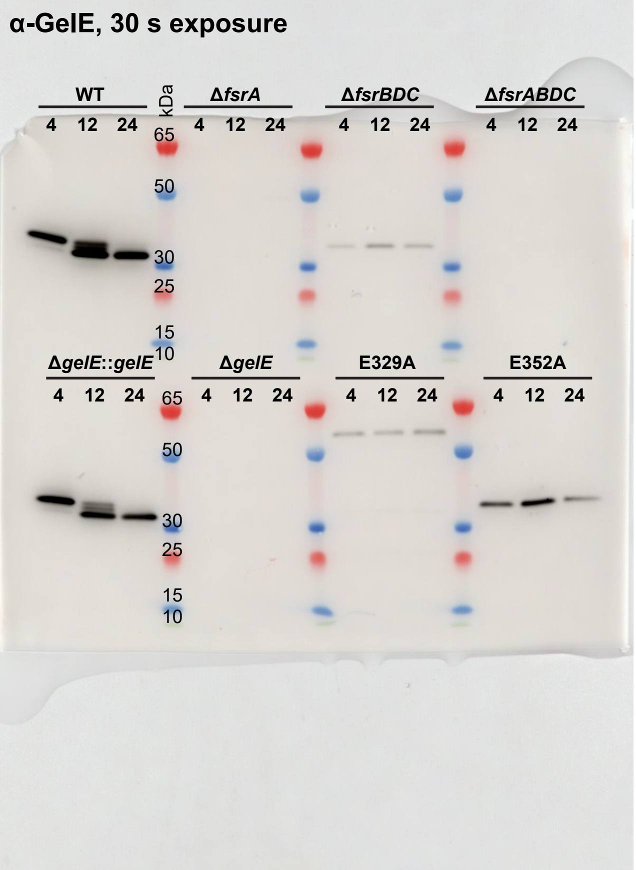

S2B Fig. Proteolytic mutants E329A and E352A show defects in extracellular secretion and autocatalytic processing of GelE.

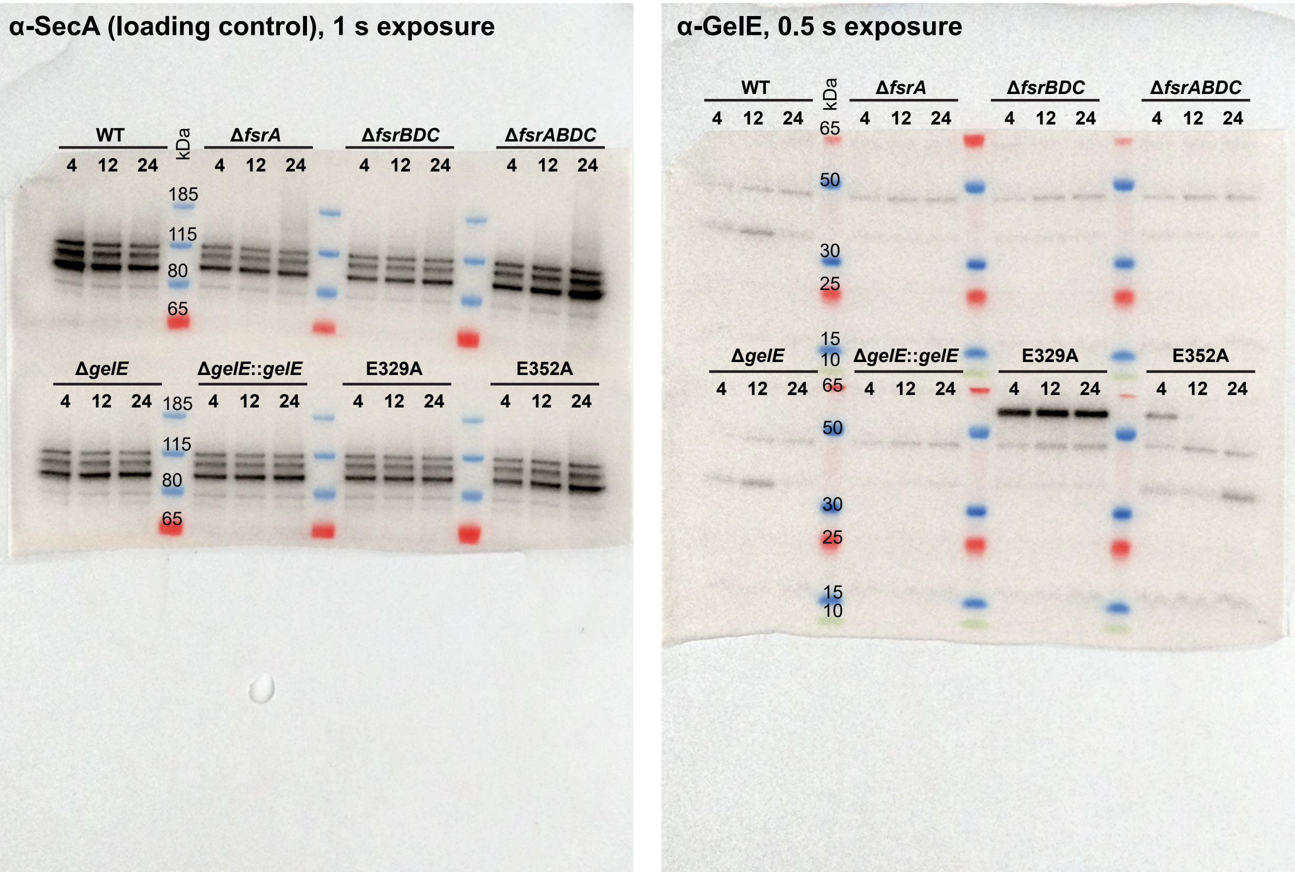

S6 Fig. Clinical *fsrABDC-gelE*+ strain EF_1008 may exhibit low-level Fsr-independent expression of gelatinase.

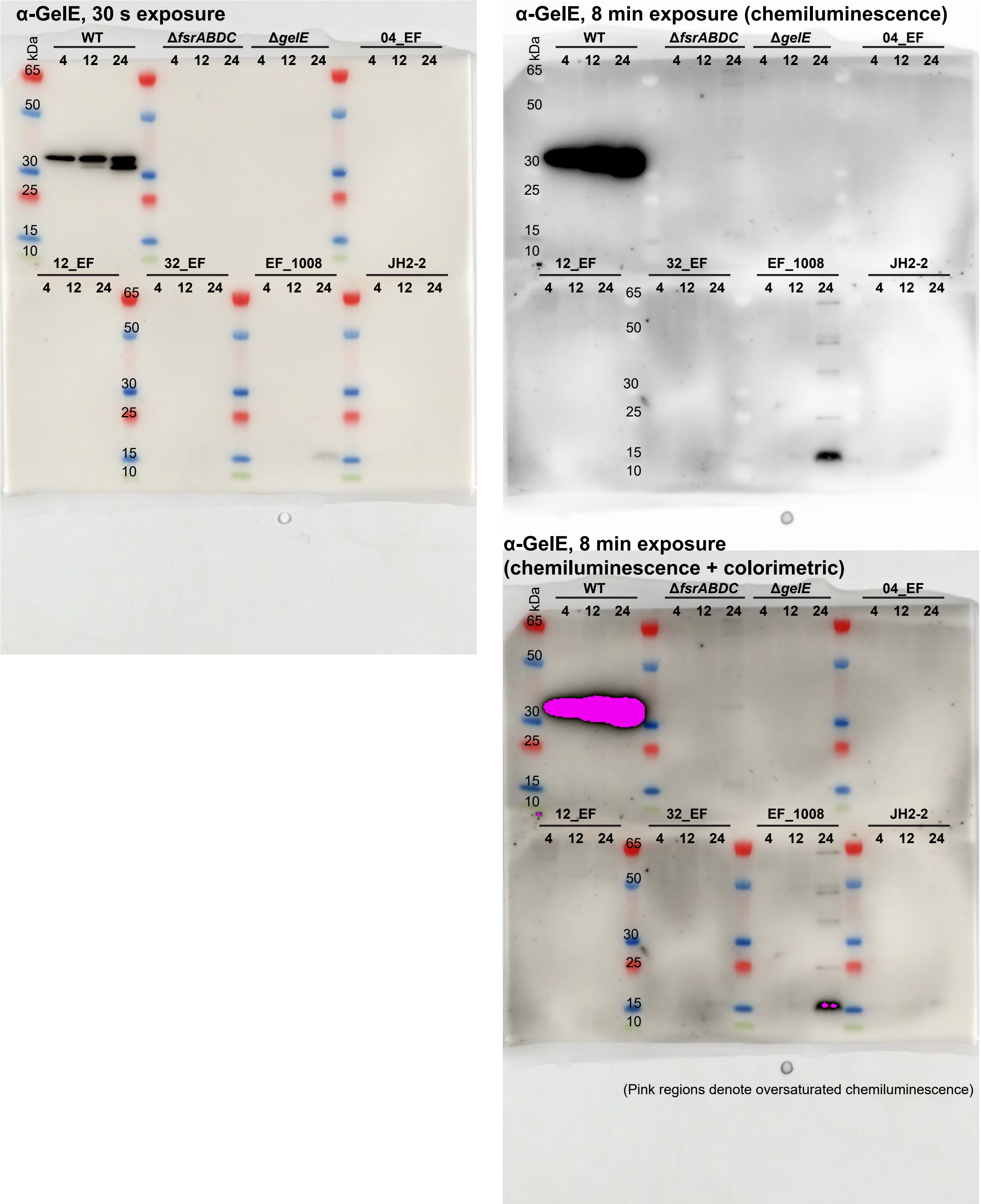

(Pink regions denote oversaturated chemiluminescence)
