# Supplementary material for: Gelatinase regulates the egress of intracellular replicating populations during Enterococcus faecalis infection
Source: PLoS Pathog. 2026 Mar 10;22(3):e1013738. doi: 10.1371/journal.ppat.1013738 (PMC12994788; doi:10.1371/journal.ppat.1013738)
Supplement: Attachment — Submitted filename: Response to reviewers_Tanoto-FR.docx [file ppat.1013738.s023.docx]

We would like to thank the reviewers for their thorough assessment and constructive feedback that helped substantiate the findings of the study. We also thank the reviewers for appreciating the novelty of the study and how it is advancing our understanding of *E. faecalis* pathogenesis and its intracellular lifestyle.

In the revised manuscript, we have introduced additional data and clarifications to:

1. Further support the direct role of GelE in regulating intracellular populations
2. Explain the phenotypic variability of across *fsrABDC-gelE+* clinical strains
3. Provide preliminary insights into the subcellular localization of replicating and non-replicating *E. faecalis*
4. Provide a comparative view of QS expression between intracellular and extracellular *E. faecalis*

Below, we provide a point-by-point response (shown in black) to all reviewer comments (shown in blue), starting with major comments raised by Reviewer #1, followed by other minor comments raised by all reviewers. Changes are tracked and indicated in red font in the revised manuscript with tracked changes.

**Major Comments:**

**Reviewer #1**:

1. Can the authors provide data or cite previous evidence to clarify whether the serine protease SprE, which is also regulated by the Fsr system, contributes to the increased intracellular survival in macrophages? It is unclear whether SprE has been sufficiently ruled out as a contributing factor to intracellular survival and replication.

We have now tested the intracellular survival of a transposon insertion mutant in *sprE* alongside the deletion mutants and observed comparable intracellular CFU to WT.

To clarify that SprE does not contribute to the modulation of intracellular survival, we have **introduced new data** **in the revised manuscript** as follows:

1. Appended the intracellular CFU data for *sprE*::Tn (n = 5) to **Fig 1D** (6 hpi), **Fig 1E** (20 hpi) as well as **S1F Fig** (2 hpi), for which there are no significant differences compared to WT at all timepoints;
2. Added the agar-based gelatinase activity assay for *sprE*::Tn in **S1G Fig** (representative of n = 3), in which we showed that *sprE*::Tn retained gelatinase activity, thus further supporting that increased intracellular CFU is specific to the absence of gelatinase secretion;
3. Updated the manuscript in lines 120-122 and lines 131-133, describing that the gelatinase-positive *sprE*::Tn showed comparable intracellular survival to WT;
4. Updated **S1 Table** (Row 12) with details on the *sprE*::Tn mutant used;
5. Relabelled **S1G Fig** (**original manuscript,** 2 hpi intracellular CFU of complementation strains) as **S1H Fig** (**revised manuscript**). All in-text references and figure legends of these respective figures have been updated accordingly.

2. Line 173-183: The authors should clarify whether the absence of fsrA and gelE, directly accounts for increased intramacrophage survival, given that all fsrABCD-gelE- strains show elevated CFU by 6 hpi. In contrast, the persistence of fsrABCD-gelE+ strains appears delayed, with elevated CFU observed only at later timepoints and to a lesser degree than the fsrABCD-gelE- strains. Could this discrepancy be explained by leaky gelE expression, in the absence of fsrABCD, at levels too low to be detected by qualitative method (i.e., gelatin agar assays)? Assessing protein expression may provide further insights into these differences among the fsrABCD-gelE+ strains and may explain why isolates 32_EF and EF_1008 fail to proliferate.

The reviewer is asking whether leaky *gelE* expression could account for the variability in intracellular survival among *fsrABCD-gelE+* strains. We have added new Western blot data showing minimal GelE secretion in EF_1008 supernatants compared to the oversaturated WT GelE bands, but no detectable GelE secretion for 32_EF. The signal in EF_1008 supernatant is detectable only after prolonged membrane exposure (8 min) and only in samples harvested at 24 h (**newly added in the revised manuscript as** **S6 Fig**). While these data indicate that low-level residual GelE secretion may occur in some *fsrABCD-gelE+* strains, leaky GelE expression does not explain the variability in intracellular CFU observed across this group. Notably, the OG1RF control strain Δ*fsrABDC* still exhibits elevated intracellular CFU at 20 hpi (Fig 2D) despite showing similar leaky GelE expression as EF_1008 (S6 Fig), indicating that this expression level is not sufficient to account for the differences in intracellular survival.

Morever, growth kinetic analysis of selected *fsrABDC+gelE+*, *fsrABCD-gelE+* and *fsrABCD-gelE−* clinical isolates (shown in Fig 2 and Fig S5) in DMEM + 10% FBS over 24 h showed that most of the *fsrABDC-gelE+* isolates, unlike Δ*fsrABDC*, exhibited slower growth compared with OG1RF control strains (**see growth curve in Figure below**; dashed black lines indicate OG1RF WT growth curve). Therefore, the reduced intracellular CFU observed for 32_EF and EF_1008 cannot be attributed to inherent growth differences.

Finally, it is interesting to point out that the baseline intracellular CFU of 32_EF in macrophages at 2 hpi is lower compared to other *fsrABCD-gelE+* strains, and thus it may explain why intracellular CFU for 32_EF does not reach the levels of Δ*fsrABDC* by 20 hpi.

Given the expected diversity in clinical strains (as inferred from their sequence types, described in S2 Table), we hypothesise that other factors within the deletion regions of *fsrABDC-gelE+* and *fsrABDC-gelE-* strains, and/or additional genomic variations in other loci may also cause differences in intracellular survival and/or replication of these clinical strains; however, such investigation is beyond the scope of this paper.

In summary, to strengthen the conclusions regarding our clinical strains, we have introduced the following **new data in the revised manuscript**:

1. Switched the order of **S2 Fig** (structure of GelE active site) with **S3 Fig** (Growth curve + GelE Western blot in cell lysates)**;**
2. Added the Western blot (low and high exposure) of supernatants of *fsrABDC-gelE+* clinical strains as **S6 Fig**;
3. Discussed briefly regarding the variability in 20 hpi intracellular CFU of *fsrABDC-gelE+* strains (lines 190-193);
4. Provided all uncropped and labelled Western blot images as separate supplementary information (**S1 Data**).

3. Line 209: It is not entirely clear whether E. faecalis is replicating within the cytosol, phagosome, or phagolysosome. Could the non-replicating cells be trapped in the phagosome, while the replicating cells have escaped? Including an additional labeling of phagosome-derived vesicle in macrophages with a membrane dye might help provide further insight into this distinction.

To distinguish between cytosolic and non-cytosolic replicating bacteria, we performed a differential permeabilization method of immunolabelling intracellular bacteria validated in previous literature (O’Neill et al, 2016, PMID 27073088; Meunier and Broz, 2016, PMID 26274778; Malik-Kale et al, 2012, PMID 22719929) combined with eFluor 670 staining to indicate their replication status: bacteria that retained eFluor 670 signal (eFluor 670^+^) had not replicated, while those that lost the signal (eFluor 670^-^) had replicated.

Using this qualitative approach, we show a heterogeneous intracellular localization and replication status across *E. faecalis* strains, with bacteria replicating in both the cytosol and non-cytosolic compartments (**newly added as S12A Fig in the revised manuscript**)*.* Although large clusters of replicating WT and Δ*gelE* *E. faecalis* at 4 and 6 hpi tended to be cytosolic (shown under ROI 2), and we did observe instances of non-cytosolic, non-replicating bacteria (indicated by white arrows), we also detected smaller bacterial clusters of replicating bacteria being non-cytosolic (indicated by yellow arrows). Thus, although it is likely that replicating intracellular *E. faecalis* may eventually be found in large clusters within the cytosol, our current data do not suggest that bacterial replication is impaired in non-cytosolic compartments.

We additionally include transmission electron microscopy (TEM) images of WT and Δ*gelE* infected macrophages at 6 hpi (**newly added as S12B Fig in the revised manuscript**), for which we observe instances of possible phagosomal escape/cytosolic localization (inferred from the lack of a single membrane encapsulating the bacteria, indicated by red arrows) as well as non-cytosolic localization (i.e. within membrane bound compartments, indicated by blue arrows) for both intracellular WT and Δ*gelE*. While the replication status of the bacteria imaged cannot be determined from the TEM images, they support our hypothesis that WT and Δ*gelE* *E. faecalis* may escape heterogenously into the cytosol by 6 hpi.

In summary, to provide these preliminary insights into the intracellular localization of replicating *E. faecalis*, we have introduced the following **new data in the revised manuscript:**

1. Added staining of cytosolic *E. faecalis* at 2, 4 and 6 hpi by differential permeabilization as **S12A Fig**;
2. Added TEM images of intracellular WT and Δ*gelE* in 6 hpi infected macrophages as **S12B Fig;**
3. Discussed briefly on the cytosolic localization of replicating intracellular *E. faecalis* for both WT and Δ*gelE*-infected macrophages (lines 309-319);
4. Added method for staining for cytosolic *E. faecalis* under the “Confocal microscopy of infected macrophages” section (lines 720-737) and TEM imaging under a new section “Transmission electron microscopy” (lines 739-755).

4. Fig. 4C: Based on the localization of GFP-expressing E. faecalis, the non-replicating cells appear punctate, whereas the replicating cells are dispersed along the periphery of the macrophages. It would be helpful if the authors could comment on whether replicating and non-replicating cells are differentially compartmentalized within macrophages.

The compartmentalization of replicating *E. faecalis* is difficult to derive from Fig 4C since it is a flattened z-stack and therefore does not convey spatial information along the Z-axis. It was primarily included to visually demonstrate that cell sorting based on the two-colour fluorescence dilution successfully separated cells containing replicating (GFP^hi^) and non-replicating (GFP^lo^) *E. faecalis*. To better showcase replicating and non-replicating *E. faecalis* within the intracellular host environment, we provide in the Figure below three representative 3-dimensional views of Z-stack imaging of WT-infected macrophages at 6 hpi and 20 hpi, one of which has been shown in Fig 3C of the original manuscript (boxed in red below). From these 3D reconstructions, we observe no clear, exclusive punctate staining of non-replicating *E. faecalis* (which can be present in clusters, as seen in 6 hpi **(B)** and **(C)**, 20 hpi **(B)**), nor peripheral clusters of replicating *E. faecalis* (which can be present as dispersed individuals within the host cells, as seen in 6 hpi **(A)**, 20 hpi **(A)** and **(C)**). In fact, the same infected macrophage may harbor both replicating and non-replicating *E. faecalis*, once again suggesting heterotypic trafficking of these intracellular populations. This is consistent with the **newly added imaging shown in S12A-B Fig in the revised manuscript** and our response to Major Question #3 from Reviewer 1.

5. Line 240: It would be helpful if the authors could examine whether the fsrABCD system is expressed in non-phagocytosed GFP-expressing E. faecalis cells. Interestingly, replicating E. faecalis (GFPhi) comprise only a small proportion (1-5%) compared to the total non-replicating cells (GFPlo) within macrophages. One possibility is that fsrABCD is highly expressed outside of host cells, and that phagocytosis acts as a bottleneck event that dampens expression. This may explain why a significant proportion of E. faecalis cells remain non-replicating and express low levels of fsr and associated effectors.

In addition, it would be informative to know the baseline expression of the fsr system during extracellular replication at 2, 6, and 20 hours (i.e., in the absence of macrophages).

Quorum sensing (QS) via the FsrABCD system is dependent on the local concentration of the GBAP (the autoinducer peptide of the Fsr QS system). In the extracellular environment, where bacterial numbers are high, GBAP is expected to accumulate rapidly, leading to robust activation of QS. We agree that phagocytosis likely represents a bottleneck event in which a small number of bacteria become isolated within a new intracellular compartment containing little or no GBAP, which would be expected to transiently dampen QS activity. However, this alone does not explain why a substantial fraction of intracellular bacteria remains non-replicating, as *fsrABDC* expression itself is not a driver of bacterial replication and all *fsrABDC* deletion strains were able to replicate intracellularly (Fig 3A-B). Instead, we propose that QS activation is a consequence rather than a prerequisite of intracellular replication: intracellular bacteria that successfully initiated replication causes gradual GBAP accumulation within the host cell, which at sufficient density leads to QS activation.

In line with this hypothesis, and to also address the reviewer’s question about baseline expression of the FsrABDC system, we have **newly included S10 Fig in the revised manuscript**. This new data shows that *fsrABDC* and its associated regulon expression is dampened early after bacterial engulfment at 2 hpi, and remains dampened in non-replicating (within GFP^lo^ macrophages) 6 hpi populations compared to extracellular bacteria. As intracellular bacteria replicate at 6 hpi (within GFP^hi^ macrophages), *fsr* regulon expression rebounds to levels approaching those observed extracellularly.

In light of this comment, the following **new data was added to the revised manuscript**:

1. Added gene expression data of the Fsr regulon in 2 hpi and 6 hpi intracellular *E. faecalis* relative to extracellular *E. faecalis* as **S10 Fig**;
2. Compared Fsr regulon expression between intracellular and extracellular *E. faecalis* in the revised manuscript Results section (lines 257-266);
3. Appended the “Cell sorting of infected macrophages” section of Materials and Methods with the culture and harvest of 6 h extracellular *E. faecalis* (lines 635-637).

6. Line 289-296: Similar to the point mentioned earlier, could the large, highly dense intracellular ∆gelE cells reflect an inability to escape from the phagosome, whereas the more dispersed bacterial clusters observed in WT cells represent successful escape?

We refer to Major Comment #3 from Reviewer 1 on the phagosomal escape of replicating populations, in which we performed cytosolic staining and transmission electron microscopy of WT- and Δ*gelE*-infected macrophages (**added as S12A-B Fig in the revised manuscript**). We found large cytosolic replicating clusters of both WT and Δ*gelE* at 4 and 6 hpi, suggesting that phagosomal escape is likely independent of GelE. However, our current microscopic approach remains preliminary and qualitative, and we cannot rule out that there may be differences in phagosomal escape dynamics of WT and Δ*gelE* between 2 to 6 hpi.

7. Line 285, Fig. 5B: The ∆fsrABDC and ∆fsrA mutants appear to egress at levels that surpass the wild-type, despite lacking secreted GelE. Do the authors have qPCR data to assess whether other fsr-regulated factors are differentially expressed in these mutants? This may help clarify whether compensatory virulence factors contribute to the enhanced egress phenotype.

We currently do not have qPCR data on Fsr-regulated factors during the timepoints of egress (6 – 12 hpi). As proposed in the manuscript, we hypothesise that other fsr-regulated factors may regulate replication and/or egress (lines 302-303). This is an interesting question that will be followed up in future studies.

**Reviewer #2:** No major issues, no additional experiments requested.

**Reviewer #3:** I don't have major issues with this manuscript.

**Minor Comments:**

**Reviewer #1:**

1. Line 74: Please define the fsrABDC locus, briefly describing the roles of fsrA, fsrB, fsrC, and fsrD to orient readers unfamiliar with their functions.

We thank the reviewer for this suggestion. The following **changes are made in the revised manuscript:**

1. Brief description of the roles of each *fsr* gene in the introduction (lines 75-77).

2. Fig. 1A, B: Please provide an annotation table of genes with their known or predicted functions. Although the genes and associated proteins are described in the introduction, including a table would improve the clarity for the readers. In particular, while the OG1RF 12401::Tn mutant disrupts the gene involved in the phosphotransferase systems PTS8, it is unclear which gene is disrupted in the 12402::Tn mutant.

To further improve clarity to the readers, we have **made the following changes to the revised manuscript:**

1. Expanded and updated the “Description” column (column 2) of **S1 Table** to include known or predicted functions of each mutated gene (rows 5-8, 12-27);
2. Edited the Results section in lines 100-101 to direct the reader’s attention to **S1 Table**, which contains the gene description for each mutant shown in Fig 1.
3. Added the gene locus tag for PTS8 (OG1RF_11399 to OG1RF_11402) to the introduction in lines 62-63.

3. Line 162: Please clarify in the text that the “fsrABDC–gelE+” strains lack fsrA but retain either an intact or partial fsrBDC.

The exact deletion junction involving *fsrC* has been highlighted in the manuscript (lines 175-176) as supported by S4A Fig (showing blastn results of nucleotide position match to *fsrC*) and S4B Fig (showing extent of genomic deletion involving *fsrA, fsrBD* and part of *fsrC*).

However, to improve clarity for the readers, we have **made the following changes to the revised manuscript:**

1. Changed description for *fsrABDC-gelE+* strains in the Results section such that it “… lacked *fsrA* and had a truncated *fsrBDC*.” (lines 171-172).

4. Fig. 3C: Microscopy images are blurry. Suggest putting in new higher resolution images.

We apologize for the blurry images in the submitted manuscript. The resolution was likely compromised during several file format conversions done prior to submission. **The relative size of Fig 3C has been increased**, and the original TIF file for Fig 3C has been submitted at full resolution to the publication team.

5. Line 274: At 6 hpi, GelE appears to may a role in host cell lysis. However, by 20 hpi, comparable cytotoxicity is observed in both GelE+ and GelE– strains, as the authors note. Could this later-stage cytotoxicity be attributed to other virulence factors that mediate macrophage lysis and bacterial release (e.g., SprE or additional proteases, cytolysin, Epxs or other pore-forming toxins)?

It is possible that later stage cytotoxicity at 20 hpi is caused by other virulence factors that cause macrophage cell death, lysis or bacterial release (although, as a caveat, the parental OG1RF is itself cytolysin-negative). However, our primary hypothesis is that at later timepoints, continuous intracellular replication in dense GelE- populations would inevitably exert physical strain to the host cell, resulting in host cell lysis and the release of these intracellular populations at later timepoints. This phenomenon remains to be verified in future studies, using live imaging of infected cells to track the fate of intracellularly replicating GelE^+^ and GelE^-^ *E. faecalis*.

6. Fig. 5A: Does complementation restore cytotoxicity to wild-type levels? It would strengthen the conclusions to include the complemented strain (∆gelE::gelE).

While we currently do not have cytotoxicity data during infected by the complemented strains, we showed in Fig 5C that the egress kinetics of Δ*gelE*::*gelE* (light blue line) is restored comparably to that of WT (black line). Since host cell death accompanies the bacterial egress reported herein, we believe that *gelE* complementation should restore cytotoxicity to WT levels.

**Reviewer #2:**

1. Clarification about GelE catalytic residues: In Fig. S2, authors state in the legend “Only mutations in homologous residues E346 and E369 of MCP-02 produced stable, non-proteolytic proteases.” Is this by AlphaFold prediction or experimentally tested (and needs a citation)? A clarification in the legend in general could be helpful because it took me a minute to figure out that the chosen mutations for GelE were based on homology to other proteases where the catalytic residues were known.

While the structure of GelE was derived by AlphaFold prediction, the site-directed mutagenesis result of various residues in MCP-02 was experimentally demonstrated by Gao et al (2010; PMID 20876133) and is detailed in their supplementary table.

**The following change has been made in the revised manuscript**:

1. The relevant citation for MCP-02 has been appended in the legend of **S3 Fig**, and the figure caption has been modified to highlight the link to this previous study.

2. Clarification about the GelE mutants: at line 130-131, it says the E329A mutant is impaired in secretion – is this because there is less protein in the supernatant compared with the supernatants in the other strains in Fig. 1J? Can a loading control be included in Fig. 1J as it was for Fig. S3? It Why are the bands different in size and intensity in Fig. 1J (more separation between pro- and processed GelE, more intense) vs Fig. S3 (less separation between pro- and +CTD, and more separation between +CTD and fully processed GelE, less intense bands)?

We thank the reviewer for this question. The finding that E329A is impaired in secretion is partially observed as lower band intensity of pro-GelE (~55 kDa) in the supernatant (Fig 1J). However, this claim can be further supported by looking into non-secreted, intracellular GelE expression in the cell lysates (S2B Fig), for which we find intense ~55 kDa pro-GelE bands in E329A cell lysates from 4, 12 and 24 h cultures (and a similar, less intense band at 4 h E352A cell lysates). Altogether, these blots suggest that pro-GelE in E329A is mostly retained intracellularly within the cell lysate, implying a defective secretion possibly due to the loss of autocatalytic activity by the catalytic E329 (Del Papa et al, 2007, PMID 17921295; also by active site homology shown in S2 Fig).

Furthermore, the reviewer asked if a loading control can be provided for secreted GelE in the supernatant (Fig 1G, 1J), similar to SecA for intracellular GelE (S3 Fig in the original manuscript; **S2B Fig in the revised manuscript**). While a stably expressed secretory ATPase SecA exists as a loading control for intracellular lysates (Afonina et al, 2020, PMID 33082254), there is no known stably secreted protein that can be used as a loading control for secreted proteins. In fact, it has been shown that *fsr/gelE* mutants can have a significantly different exoproteome compared to WT (Shankar et al, 2012, PMID 22428053). Because we cannot normalize the secreted fraction to a stably expressed secreted protein, we ensured that all supernatants were collected from cultures of the same volume, comparable growth phase and comparable bacterial density across all timepoints and strains. A growth curve for all strains tested in Fig 1G, 1J is **newly included as** **Fig S2A in the revised manuscript**, for which all cultures showed comparable absorbance at 600 nm (Abs_600_) readings and therefore culture density at 4, 12 and 24 h.

The reviewer also asked about differences in the lower molecular weight bands (< 50 kDa) of Fig 1J and S2B Fig. We would like to clarify that in S2B Fig, the lower intensity bands at ~50 kDa and ~35 kDa from the cell lysate are likely non-specific, since these bands appear in Δ*gelE* cell lysates which cannot express GelE. To clarify this point to the readers, we have **annotated these non-specific bands in S2B Fig**, and also made the uncropped Western blot image available as Supporting Information.

Based on this minor comment, we have introduced the following **new data and changes in the revised manuscript**:

1. Added growth curve of deletion and complementation strains as **S2A Fig**, with culture timepoints of 4 h, 12 h and 24 h highlighted;
2. Relabeled S3 Fig in the original manuscript (Western blot of intracellular GelE) as **S2B Fig**.
3. Cited **S2A Fig** and highlighted that culture supernatants were “collected at different growth timepoints” (lines 126-127), which is also now reiterated under “Western blot” in Materials and Methods (lines 561-562).
4. Labelled ~50 kDa and ~35 kDa non-specific bands in **S2B Fig.**

3. The results section for Fig. 4 needs a little more narrative explanation. For example, it needs to be more explicitly stated that these experiments were performed on a WT strain with GelE expression and therefore has a lower intracellular replication rate than mutants discussed earlier. I was surprised that the number of cells with replicating bacteria were so low until I realized this. I was also confused about why gene expression was not different at 20hrs. Are the authors implying that the bacteria in the GFP-lo cells (which are called non-replicating in the results section, supported by data in Fig. 1) upregulated GelE independent of quorum sensing? Or that at 20 hr p.i. there's no additional replication in the GFP-hi cells that upregulated quorum sensing?

We thank the reviewer for the critical reading of this section and we apologize for the confusion. Here, we would like to clarify that we do not claim a lower intracellular replication rate for GelE^+^ strains (e.g. WT OG1RF, which is used in Fig 4); rather, because intracellular GelE^-^ strains continue replicating without GelE mediated egress, the replicating population remains intracellular for longer and thus a greater proportion of eFluor- (replicating) bacteria can be found in the macrophage lysates (Fig 3B).

Next, we would like to address the reviewer’s question on why gene expression is not different between intracellular replicating (within GFP^hi^ macrophages) and non-replicating populations (within GFP^lo^ macrophages) at 20 hpi. By comparing the relative expression levels in the GFP^lo^ population at 6 hpi (Fig 4D) and 20 hpi (Fig 4E), we can see that the expression of *fsrABDC* and its regulon remain rather stable between 6 and 20 hpi. On the other hand, in the GFP^hi^ population, relative expression levels for the same genes start to drop between 6 and 20 hpi. One interpretation of this result is that QS gene expression is not constant, but rather peaks prior to the eventual bacterial egress. Similar QS dynamics have been shown in an experimental infective endocarditis model (Antypas et al, 2026, PMID 41535299), in which *fsrC* expression peaked at 24 hpi and subsequently dropped at 72 hpi, resulting in the Fsr-dependent regulation of biofilm growth on heart valves only at 72 hpi. We also cannot rule out the possibility that the observed decrease in gene expression is associated with slowed bacterial replication in the 20 hpi GFP^hi^ population.

To minimize potential misconceptions to the readers as raised by this reviewer, we have **made the following changes in the revised manuscript**:

1. Updated manuscript in lines 244-245, 248 and **Fig 4 caption** to clarify that all experiments in Fig 4 are performed with WT OG1RF;
2. Edited title of the relevant Results section (line 205), **Fig 3 caption** and writeup in lines 224-225, 228 to reduce the misconception that GelE- strains have increased replication rate.

4. In Fig. 5B and 5C, do the authors have an explanation for why the mutants lacking gelE have more (5B) or an equal (5C) number of egressed bacteria at the late time points? Is the high cytotoxicity (and presumably lysis) at the late time points indicated in Fig. 5A an alternative avenue of egress? Address in discussion?

The higher numbers of egressed bacteria observed in Fig 5B pertains to the *fsrA* mutants (Δ*fsrA* and Δ*fsrABDC*) and therefore could suggest that additional *fsr*-regulated factors are regulating egress.

At late timepoints, as mentioned in a response to minor comment #5 from Reviewer 1, it is possible that replicating intracellular GelE- populations cause continuous physical strain to the host cell, and inevitably caused the host cell to lyse.

5. Would like a little more elaboration or reorganization in the discussion about selection pressures in the wound site (lines 355-362, 383-400). There were no fitness advantages in the mouse model (Fig. 6), but this is in the absence of antibiotic treatment of the mouse and other human-specific skin microbes that may be present in natural infection, which might be able to apply such selection pressure. Furthermore, are there dis/advantages to dysregulating the expression of other fsr-regulated genes, such as the ones that resulted in lower bacterial recovery shown in Fig. 1? The authors suggest the role of biofilms later on (lines 393-395). Would the combined effect help put pressure on mutating the fsr locus? Or since later in the discussion the authors state that fsr locus and gelE loss are common among isolates from other infection sites, does the mutation occur elsewhere in the body and confer an advantage in a skin wound site? Is it surprising that there’s no clonal expansion of these mutants?

We agree with the reviewer that perhaps a different/additional selection pressure relevant to clinical infection, such as antibiotic treatment or later timepoints, would be required to highlight the colonization fitness of GelE- strains during infection. We have revised the discussion to highlight that future experiments could focus on investigating the increased intracellular reservoirs in wound under selective pressure (lines 426-430).

Other Fsr-regulated genes, such as *sprE* (Qin et al, 2000, PMID 10768947) and *entV* (Teixeira et al, 2013, PMID 23734216), as well as bacterial proteolytic targets of GelE such as Ace (Lebreton et al, 2009, PMID 19433548), have been implicated as virulence factors in other infection models, but not for wound infection. Our work shows that these Fsr/GelE-regulated factors do not exhibit greater intracellular survival *in vitro* (Fig 1A-B). However, it remains to be fully investigated whether their absence modulates pathogenesis in wound infection. In a previous transposon sequencing analysis of *E. faecalis-*infected wounds, transposon insertion mutants in *atlA*, *entV*, *fsrB* and *fsrA*, but not *gelE*, were significantly less abundant in 3 dpi wounds compared to the pre-inoculation mutant pool (Tan et al, 2023, PMID 38078746, Supplementary File 1), suggesting that additional Fsr/GelE-regulated factors may play a role in conferring persistent wound infections independent of GelE activity.

We have also elaborated on how genomic deletions of *fsrABDC* and *gelE* might occur (lines 380-382, 418-423). While the prevalence of *fsr/gelE* loss in clinical isolates has been reported in numerous studies (Antypas et al, 2026, PMID 41535299; Li et al, 2025, PMID 40444324; Galloway-Peña et al, 2011, PMID 21097591; Nakayama et al, 2002, PMID 12039782), the selection pressure for this genetic mutation remains unknown. Most recently, the infective endocarditis study by Antypas et al (2026, PMID 41535299) tracked patient isolates longitudinally and found no emergence of *fsr/gelE* deletion nor revertants of *fsr/gelE* mutants in all patient samples studied (see Supplementary Table S3 of Antypas et al). Clonal expansion of the *fsrABDC-gelE+* strains has been proposed in several previous studies as a mechanism driving its prevalence in clinical isolates; however, since these *fsrABDC-gelE+* strains are of different sequence types (as reported in previous studies; also in S4A Fig, S2 Table of this study), it is also likely that *fsrABDC* loss occurred independently in different lineages due to unknown evolutionary pressures.

Moreover, given that GelE+ and GelE- *E. faecalis* are equally fit during our competitive wound infection model (Fig 6B), it is expected that there is no clonal expansion of one strain over the other in the wound niche. Whether an overrepresentation of GelE- strains could occur in other competitive infection models, whether a selection pressure for *fsr/gelE* mutants is present in other infection sites, and whether specific genetic determinants are required for *fsr/gelE* loss in *E. faecalis* are all interesting questions for future follow-up studies.

Based on this minor comment by the reviewer, we have **updated the following Discussion points in the revised manuscript:**

1. Lines 426-430: Suggestion for possible selection pressures to investigate fitness advantage(s) of GelE- strains during wound infection;
2. Lines 380-382, 418-423: Discussion into possible mechanisms underlying the genomic loss of *fsrABDC/gelE* in clinical isolates

6. Scales on some of the figures (e.g. Fig. 1K, L, Fig. 5A-C) could be adjusted so that difference at 6 hrs p.i. are easier to see.

We thank the reviewer for this suggestion. To improve visibility of the graph and highlight the differences between strains, we have **made the following changes to the revised manuscript:**

1. Shrunk the y-axis scales of **Fig 1K-L** and **Fig 5A-C**, thus better highlighting the differences in intracellular CFU counts, cytotoxicity at 6 hpi and the egressed CFU counts respectively.
2. Accordingly, we have also adjusted the y-axis scale of **Fig 1D-E** to be consistent with that of Fig 1K-L, and **S11B-C Fig** to be consistent with that of Fig 5B-C.

**Reviewer #3:**

1. The authors show that GelE-negative strains exhibit enhanced intracellular replication (Fig. 3A–C). Mechanistically, however, the connection between GelE, the Fsr system, and replication remains unclear. Why do strains lacking these genes replicate more rapidly? Is there a metabolic or biosynthetic burden associated with producing these factors that constrains growth, or does overexpression of these systems actively slow replication? It would be helpful for the authors to elaborate on these possibilities in the Discussion and to propose future experiments that could directly test these ideas.

We would like to clarify two points raised by this reviewer comment: one, that strains lacking the Fsr system and/or GelE do not replicate more rapidly (as shown in S8 Fig); and two, that we are not proposing (though not outright rejecting) a role for GelE and/or Fsr-regulated genes in modulating intracellular replication itself. Rather, here we propose that strains that are phenotypically GelE-negative were unable to regulate the egress or exit of intracellular bacteria from the host cells after intracellular replication has occured. As a result of this dysregulation of egress, there is an accumulation of intracellularly replicating bacteria, which results in significantly higher proportions of replicating bacteria intracellularly (Fig 3A-C) and created dense intracellular populations of replicating, egress-defective bacteria (Fig 5C).

As addressed for minor comment #3 from Reviewer 2, we have revised the text in lines 244-245, 248, as well as the section title in line 208 and **Fig 3 caption** to minimize any possible misconceptions on the relationship between FsrABDC/GelE and intracellular replication.

2. Line 238 — I’m confused by the principle of the two-color fluorescence dilution FACS assay. Shouldn’t the eFluor signal decrease through dilution as cells replicate, similar to the assay described in the previous section? Why do the authors state that it remains constant? Please clarify.

Here, we would like to make a distinction between the eFluor signal observed from a single bacterium, versus that observed from a bacteria-infected macrophage (see diagram below).

Observed from a single bacterium (green boxed inset), the eFluor signal intensity indeed decreases during replication. Bacteria are pre-loaded with the eFluor dye and as bacteria divide, the eFluor signal will be distributed equally between the 2 daughter cells. Thus, with every bacterial cell division, eFluor signal intensity on the individual bacteria decreases.

However, this is different when observed from an infected macrophage (2 hpi and 6 hpi boxes). An infected macrophage only becomes eFluor+ during the initial engulfment of eFluor+ bacteria (i.e. at 2 hpi). As the bacteria replicates intracellularly (at 6 or 20 hpi), this initial eFluor signal is simply re-distributed to daughter cells, but the accumulative eFluor signal of this intracellular population—in other words, the eFluor signal observed from the macrophage—remains constant. Thus, the infected macrophage displays a constant eFluor signal while the bacteria inside it replicates (see also calculations below using arbitrary fluorescence units in RFU).

For a similar approach using plasmid-based fluorescence dilution, the reader is directed to Saliba et al (2016; PMID 27841856), from which this cell sorting method was adapted.

3. The findings on GelE’s role in mediating E. faecalis egress from infected cells are fascinating, particularly given the implication that GelE may act on one or more host-derived substrates. That said, the impact of infection with the different strains on the host cells feels like the most missing piece of this study. While I don’t think host-focused experiments are necessary at this stage, the Discussion would be strengthened by outlining plausible host substrates that could be targeted by GelE, and by briefly describing how the authors would approach testing these candidate targets in future experiments.

We thank the reviewer for this comment. Based on parallels to other host substrates during pathogenic protease-driven egress, we infer that there are multiple substrates that could be targeted by GelE (lines 394-406). Since we hypothesize that GelE could be acting on similar substrates, we would approach this investigation similarly to those studies – for instance, we can visualize the proteolytic cleavage of these reported host substrates during GelE+ infection via Western blot, as well as verifying the cellular phenotypes arising as a result of this cleavage, including induction of cell death, cytoskeletal rearrangements and/or host cell permeabilization. The manuscript discussion has been revised to highlight future approaches to investigate GelE-mediated host-pathogen interactions (lines 407-409).

Minor comment:

4. Line 105: state what the genes encode for clarity

We thank the reviewer for the feedback. The genetic mutants screened in Fig 1A-B have been briefly described in the introduction and **S1 Table**. To further improve clarity, as was addressed in minor comment #2 from Reviewer 1, we have now updated the introduction (lines 62-63, 75-77), the “Description” column of S1 Table (rows 5-8, 12-27), as well as the reference to S1 Table in the Results section (lines 100-101).
